# Structural Insights into the Diversity and DNA Cleavage Mechanism of Fanzor

**DOI:** 10.1016/j.cell.2024.07.050

**Published:** 2024-08-28

**Authors:** Peiyu Xu, Makoto Saito, Guilhem Faure, Samantha Maguire, Samuel Chau-Duy-Tam Vo, Max E. Wilkinson, Huihui Kuang, Bing Wang, William J. Rice, Rhiannon K. Macrae, Feng Zhang

**Affiliations:** (1) Broad Institute of MIT and Harvard, Cambridge, MA 02142, USA; (2) McGovern Institute for Brain Research at MIT, Cambridge, MA 02139, USA; (3) Department of Brain and Cognitive Science, Massachusetts Institute of Technology, Cambridge, MA 02139, USA; (4) Department of Biological Engineering, Massachusetts Institute of Technology, Cambridge, MA 02139, USA; (5) Howard Hughes Medical Institute, Cambridge, MA 02139, USA; (6) Cryo-Electron Microscopy Core, NYU Grossmanew York University School of Medicine, New York, NY 10016, USA; (7) Department of Cell Biology, NYU Grossmanew York University School of Medicine, New York, NY 10016, USA; (8) Equal contribution; (9) Lead Contact

**Keywords:** Fanzor, eukaryotic RNA-guided DNA endonuclease, gene editing, DNA cleavage mechanisms, structural diversity, activation mechanisms, catalytic site, R-loop structure

## Abstract

Fanzor (Fz) is an ωRNA-guided endonuclease extensively found throughout the eukaryotic domain, with unique gene editing potential. Here we describe the structures of Fzs from three different organisms. We find that Fzs share a common ωRNA interaction interface, regardless of the length of the ωRNA, which varies considerably across species. The analysis also reveals Fz’s mode of DNA recognition and unwinding capabilities as well as the presence of a non-canonical catalytic site. The structures demonstrate how protein conformations of Fz shift to allow binding of double-stranded DNA to the active site within the R-loop. Mechanistically, examination of structures in different states shows that the conformation of the Lid loop on the RuvC domain is controlled by the formation of the guide/DNA heteroduplex, regulating the activation of nuclease and DNA double-stranded displacement on the single cleavage site. Our findings clarify the mechanism of Fz, establishing a foundation for engineering efforts.

Fanzor (Fz) is a eukaryotic programmable ωRNA-guided endonuclease ^1,2^. Fzs and the prokaryotic CRISPR-Cas12 systems evolved from distinct variants of TnpB, a widespread prokaryotic Obligate Mobile Element-guided Activity (OMEGA) system ^1–7^. Like Cas12, there is significant diversity in the Fz family. Fzs are mainly found in protostomia, algae, fungi, and unicellular eukaryotes and are grouped into two distinct families: Fz1 and Fz2. Fz2 exhibits strong structural similarities at the protein level to TnpB, whereas Fz1 is considerably larger, hinting at potentially different functions and mechanisms ^2^.

To explore this diversity at the structural and functional level, we resolved the cryo-EM structures of three Fz1 proteins from different species spanning two kingdoms of eukaryotes: *Guillardia theta* (GtFz1) (algae), *Spizellomyces punctatus* (SpuFz1) (fungus), and *Parasitella parasitica* (PpFz1) (mycoparasite). Fz1s were captured in various conformational states, creating a collection of 13 structures. These structures reveal both common and distinct aspects of ωRNA binding and DNA recognition, offering insights into the diverse mechanisms by which Fz1 proteins achieve specificity and efficiency in their endonuclease activity. The examination of different conformational states sheds light on the activation and cleavage mechanisms of Fz1 proteins, elucidating the structural transitions that facilitate their function as RNA-guided endonucleases.

## RESULTS

### Structures of GtFz1, SpuFz1, and PpFz1 complexes

We previously showed that GtFz1 and SpuFz1 have RNA-guided DNA cleavage activity in vitro, with transposon-associated motifs (TAMs) 5’-CATA-3’ and 5’-TTAA-3’, respectively ^2^. For PpFz1, we conducted a TAM screen and identified a 5’-ATN-3’ TAM, confirming its ability to cleave DNA in vitro (see Methods). To investigate the structural diversity of Fzs, we expressed the GtFz1, SpuFz1, and PpFz1 ribonucleoproteins (RNPs) in *Saccharomyces cerevisiae*, purified them and determined the structures (with and without target DNA) by single particle cryo-EM (Figure S1, Table S1). We determined the structures of the GtFz1-ωRNA binary complex at resolution of 4.7 Å, a GtFz1-ωRNA-target DNA ternary complex with a target DNA containing TAM duplex and a single-strand TS at resolution of 3.3 Å, a GtFz1-ωRNA-target DNA ternary complex with full double-strand target DNA at resolution of 3.0 Å, respectively (Figures S1A–G). For SpuFz1, we aimed to capture different R-loop binding states by using four different DNA substrates in which phosphorothioate modifications had been introduced, rendering them uncleavable: partial modification of the DNA target strand (TS), partial modification of the non-target strand (NTS), complete modification of the TS, and complete modification of the NTS. This approach allowed us to capture six SpuFz1 complex structures in different DNA binding states, with resolutions ranging from 2.9 Å to 3.4 Å (Figures S1H–J). We obtained density maps of the PpFz1-ωRNA-DNA complex in four states, with resolutions ranging from 3.2 Å to 3.5 Å (Figures S1K–M).

**Figure 1.**
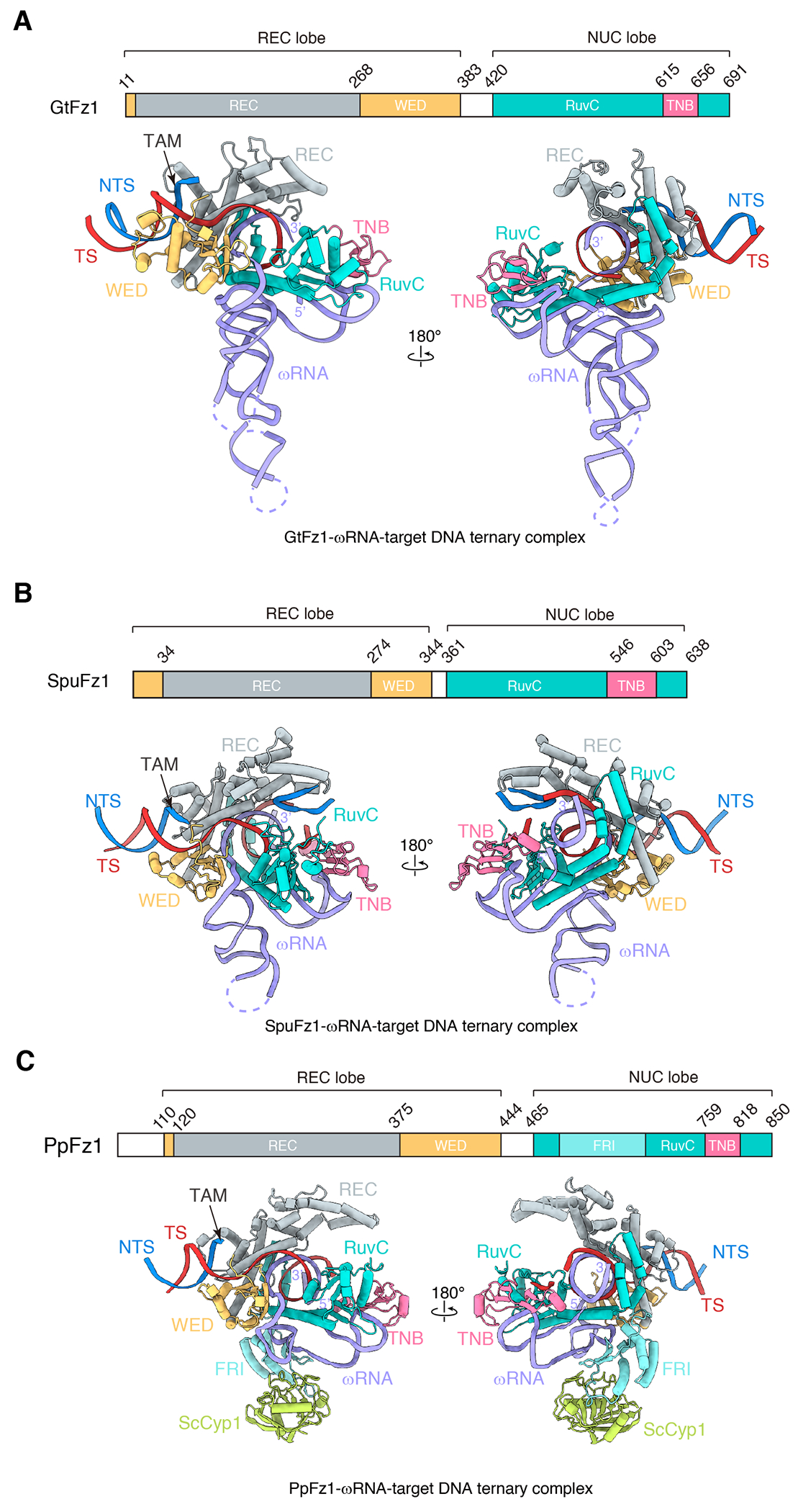
Structural overview of Fanzor1 complexes (**A-C**) Schematic locus and cryo-EM structure of the Fz1-ωRNA-target DNA complex from *Spizellomyces punctatus* (SpuFz1) (A), *Guillardia theta* (GtFz1) (B), and *Parasitella parasitica* (PpFz1) (C). REC domain is colored gray, WED domain is colored yellow, RuvC domain is colored cyan, TNB domain is colored pink, Fanzor RuvC Insertion (FRI) domain is colored light cyan; *Saccharomyces cerevisiae* Cyclophilin1 (ScCyp1) is colored green, ωRNA is colored purple, DNA target strand (TS) is colored red, and DNA non-target strand (NTS) is colored blue.

The overall structures of the three Fz1 proteins show notable similarities, all featuring a bilobed architecture and accommodating the ωRNA and target DNA in comparable ways (Figure 1), consistent with TnpB structures ^8,9^. The REC lobe, encompassing the REC and WED domains, clamps down on the DNA TAM duplex. A large cleft is formed by the REC lobe and NUC lobe, which contains the RuvC nuclease and target nucleic acid binding (TNB; previously referred to as Nuc) domains. The RNA-DNA heteroduplex is accommodated by a positive trench deep within the cleft. The interface by which the protein interacts with the RNA is similar across all three Fz1s, suggesting a conserved RNA-guided DNA-targeting mechanism among different Fz1s (Figure 1).

**Figure 2.**
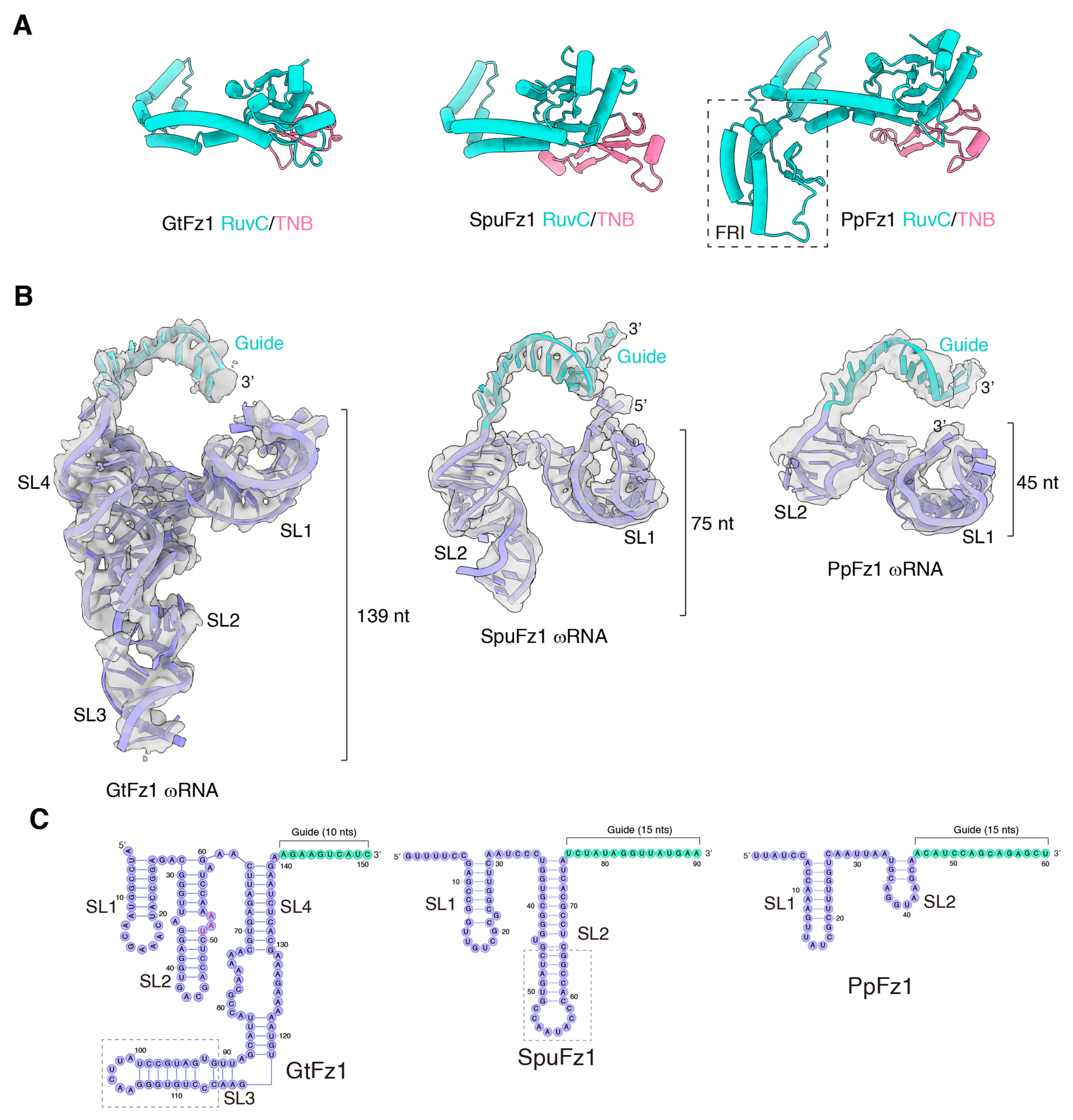
Structural diversity of Fanzor1 (**A**) Structural comparison of the RuvC and TNB domains across GtFz1 (left), SpuFz1 (middle), and PpFz1 (right). The dashed box indicates the FRI domain of PpFz1. (**B**) Comparative analysis of ωRNA structures between GtFz1, SpuFz1, and PpFz1. EM density is shown transparently. The ωRNA scaffold is colored purple, and the guide is green. (**C**) Schematic of the ωRNA in GtFz1, SpuFz1, and PpFz1.

The majority of Fz1 proteins are between 600 and 700 amino acids ^2^ and have relatively conserved domain organization and structure (Figure 1). A notable exception is PpFz1, which features an insertion of approximately 100 amino acids within its RuvC domain, which we refer to as the Fanzor RuvC Insertion (FRI) domain (Figure 1C and Figure 2A). The structure of the PpFz1 ternary complex reveals that this insertion constitutes a small domain comprising three helices, three beta-sheets, and an extensive loop (Figure 2A). Intriguingly, an unidentified density, resembling a small protein, was observed binding to the loop of the FRI domain. To identify this protein, we extracted the density from the overall structure and conducted unbiased structural matching against the yeast proteome, encompassing 6,039 proteins (STAR Methods and Methods S1). This analysis identified a match with a subset of cyclophilins (Cyps) (Figures S2A, B), a family of peptidyl-prolyl cis-trans isomerases with highly conserved structures ^10^. Mass spectrometry analysis of the PpFz1 ternary complex identified Cyp1, but not other yeast Cyp proteins (Table S2). Based on this result, we built the model of *S. cerevisiae* Cyp1 into the structure of the PpFz-ωRNA-DNA complex (Figure 1C and Figure S2B). The model shows that the active pocket of Cyp1 engages the loop in the FRI domain of PpFz1 (Figure S2C), with residues P614 and P616 from PpFz1 forming hydrophobic interactions with A99, F111, and F58 of Cyp1 (Figure S2D). Furthermore, residue K80 of Cyp1 forms a charged interaction with U26 of the ωRNA scaffold (Figure S2C). Pull-down experiments with the PpFz1 FRI domain with 11 cyclophilin homologs from *S. cerevisiae, P. parasitica,* and *Homo sapiens* showed that PpFz1 binds to a range of cyclophilins across species but has some selectivity (Figure S2E). Sequence alignment of the 11 cyclophilins reveals a highly conserved sequence, including the residues that interact with the FRI domain, suggesting that the FRI domain could bind to different homologs of cyclophilins (Figure S2F).

### ωRNA structure and interaction with Fz1

Fzs from different species have significantly different ωRNA lengths ^1,2,5^. For example, GtFz1 ωRNA is 150 nucleotides (nts), SpuFz1 ωRNA is 90 nts, and PpFz1 is 75 nts (Table S3) ^2^. Despite these substantial differences, however, all Fz1s exhibit a relatively uniform ωRNA binding interface, mainly comprising two parts (Figure S3A). The first is the surface of the RuvC/TNB domains distant from the REC domain, and the second is the junction between the RuvC and WED domains (Figure 2B and Figures S3A–C). The ωRNA structures show that the first stem loop (SL1) of the GtFz1, SpuFz1, and PpFz1 ωRNAs consistently contains seven base pairs (Figure 2C). Across all three Fz1 structures, SL1 adopts a similar conformation (Figure 2B and Figure S3A) and interacts with the same protein interface, the RuvC and TNB domains (Figure S3B). This conservation implies that the first stem loop may play an important role in the function of Fz1 across different organisms. Interestingly, however, the orientation of the 5’ ends of ωRNAs differs across these three species. The 5’ end of the GtFz1 ωRNA points outward from the core of the complex, whereas those of the SpuFz1 and PpFz1 ωRNAs face towards a large cleft formed by the protein (Figure S3D). This difference may be due to the smaller TNB domain of GtFz1, which does not provide stabilization for the 5’ end of the ωRNA.

In the scaffold region between SL1 and the 3’ end of the guide sequence, the three Fz1 ωRNAs display different structures (Figures 2B–C). The GtFz1 ωRNA structure in this region contains three stem loops, with the distal and loop regions of SL2 forming extensive interactions with SL4, folding together (Figure 2B). However, SL3 does not interact with other parts of the ωRNA or the protein; its structure is distant from the core of the complex, and its loop and distal base pairs are disordered in the structure (Figures 2B–C). In the SpuFz1 and PpFz1 ωRNA structures, there is only one stem loop, SL2, between SL1 and the guide (Figures 2B–C). PpFz1 has the smallest known ωRNA scaffold, with only four base pairs in its ωRNA SL2 in the structure, similar to the shortest engineered SpuFz1 ωRNA structure (Saito et al., 2023). These results suggest the region beyond SL1 is not critical for Fz1 function and may not be constrained during evolution.

All three Fz1 complexes exhibit conserved structures at the junction of the ωRNA scaffold and guide (Figure S3A). In each structure, the four beta-sheets of the WED domain position the first three nts of the guide sequence, while the fourth to eighth nts of the guide sequence are bound to the REC domain (Figure S3C). This interaction mode between the protein and the RNA guide segment is conserved in TnpB ^8,9^ and Cas12 family effectors including Cas12a ^11^, Cas12b ^12^, Cas12c ^13^, Cas12e ^14^, Cas12i ^15^, and Cas12l ^16^, despite the diversity in the overall structures of the WED and REC domains, indicating this mode of recognition of the guide RNA may be a hallmark of TnpB and its derivative systems.

### Mechanism of target DNA recognition

**Figure 3.**
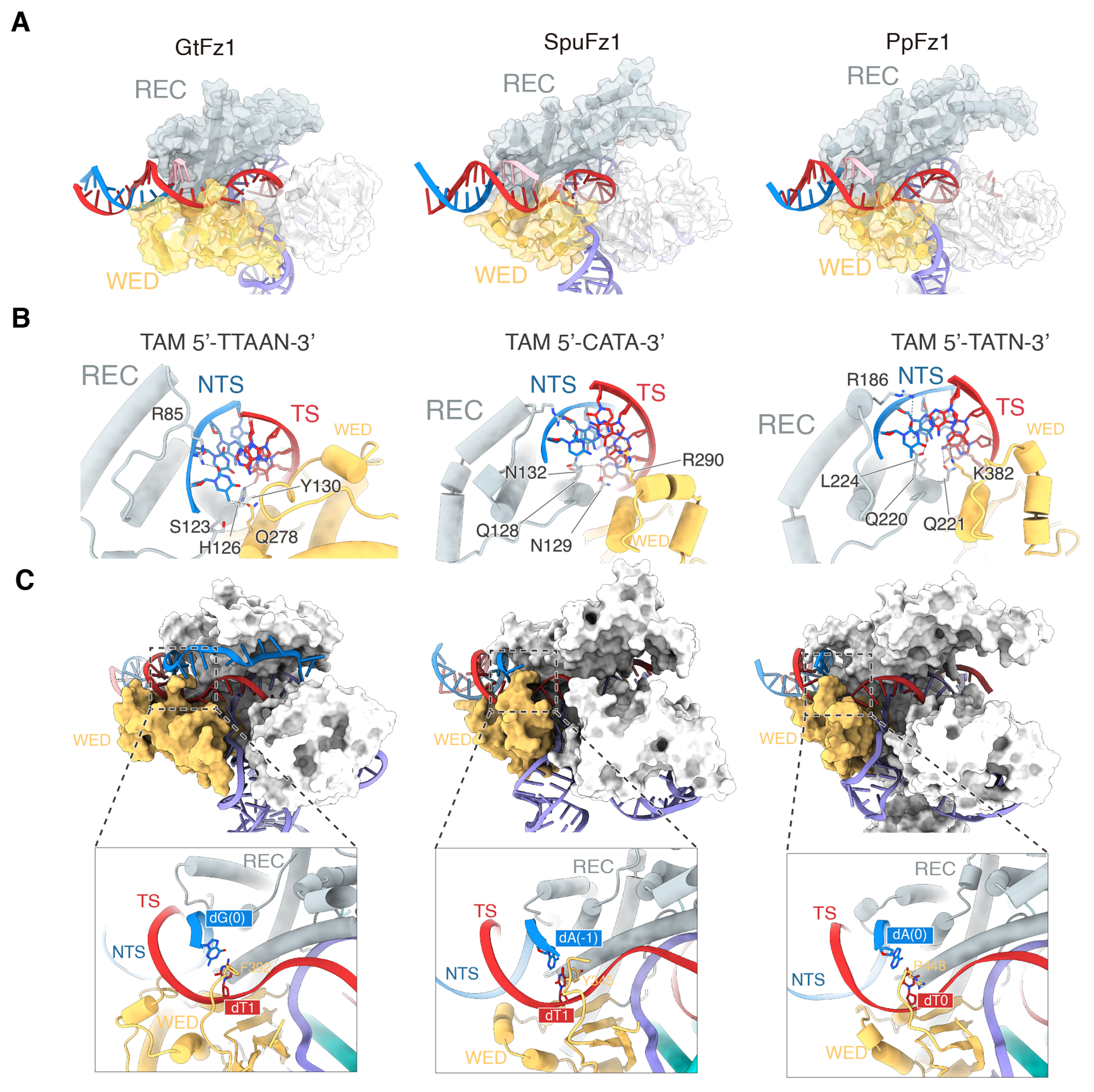
Comparative analysis of DNA recognition by Fanzor1 (**A**) Recognition of the TAM duplex by GtFz1 (left), SpuFz1 (middle), and PpFz1 (right). The TAM sequence is highlighted in pink. (**B**) Interactions involved in TAM recognition, showing a conserved structural feature among GtFz1, SpuFz1, and PpFz1. Specifically, an arginine (R) residue from a loop in the REC domain inserts into the groove of the TAM duplex. The N-terminal end of the alpha-4 helix from the REC domain, along with a short helix from the WED domain, recognizes the TAM base groups in a similar orientation. Different Fz1 proteins utilize non-conserved residues to recognize different TAM sequences. (**C**) Initiation of the R-loop by the loop from the WED domain. Protein domains and ωRNA are colored as in Figures 1 and 2, the DNA target strand (TS) is colored red, and the DNA non-target strand (NTS) is colored blue.

The three Fz1s recognize target DNA similarly, reflected in the shared recognition of the TAM region, DNA unwinding mechanism, and heteroduplex recognition. Fz1s recognize the DNA TAM duplex through a combined effort of the WED and REC domains (Figure 3A and Figure S4). A conserved arginine (R) residue, located on a loop of the REC domain, anchors into the groove of the DNA TAM duplex, providing non-base specific stability to the DNA (Figure 3B). We introduced an R85A mutation into GtFz1 and then performed a DNA cleavage assay, observing complete loss of activity, indicating this conserved R residue is critical for DNA binding (Figure S4D). The sequence specificity of the TAM is determined by interaction with the DNA and the ɑ4 of the REC domain and WED domain. Mutating the residues in GtFz1 that form interactions with TAM base groups, including S123A, H126A, Y130A, and Q278A, strongly reduced cleavage activity (Figure S4D). Furthermore, GtFz1 showed no cleavage activity for target DNA variants in which each base of the 5’-TTAA-TAM was mutated, supporting our structural observations (Figure S4E). Although the WED domain of all three Fz1s has conserved beta-sheets, the remaining parts of the WED domain structure differ substantially, including the helix providing interactions with the TAM (Figure S4B). This portion consists of 73, 29, and 28 amino acids in GtFz1, SpuFz1, and PpFz1, respectively, and lacks sequence conservation, resulting in clear differences in the interaction patterns with the TAM sequence. This variable region of the WED domain may evolve to enable recognition of diverse DNA sequences.

Following target DNA recognition and binding, the DNA duplex is unwound for Cas9 and Cas12a ^17 18–20^. In Fz1s, the loop region between the WED and RuvC domains interacts with the region between the TAM duplex and the guide/DNA heteroduplex (Figure 3C). Specifically, residue F392 in GtFz1, Y345 in SpuFz1, or R448 in PpFz1 forms a stacked interaction with the last base of the target strand (TS) in the TAM duplex (Figure 3C). This interaction stabilizes the unwound DNA state and allows the TS to base pair with the guide. Mutating F392 to alanine in GtFz1 significantly reduced DNA cleavage activity (Figure S4F). To test the tolerance of Fz1 to mismatches between the guide sequence and target DNA, we performed cleavage assays with target DNA variants containing single mutations from dA(−1) to dG(−19), finding that only the 3^rd^ bp mismatch significantly reduced activity (Figure S4G). Correspondingly, the structure of GtFz1 shows that the 3^rd^ guide/DNA bp is stabilized by ɑ4 of the REC domain by forming interactions with residues T137 and N141 (Figure S4H).

The guide/DNA heteroduplex is 9-bp long in GtFz1 and 15-bp long in both SpuFz1 and PpFz1 (Figure 3A). To understand this difference between GtFz1 and SpuFz1/PpFz1, we aligned the GtFz1 and SpuFz1 proteins and discovered that alpha helices 5 and 6 of the SpuFz1 RuvC domain interact with 6 bps at its end, a region where GtFz1 and SpuFz1 show conformational differences. Specifically, the two helices in GtFz1 are shifted towards the interior of the complex, creating steric hindrance for the last few bps of the DNA (Figure S4I). This suggests that the conformation of ɑ5 and ɑ6 at the end of the RuvC domain governs the number of bp of heteroduplex the protein can accommodate. This may explain why SpuFz1 preferentially binds to longer heteroduplexes than GtFz. We observed a small fraction of the SpuFz1 particles (1.8%) with extended heteroduplex density, a variant we classify as State II (Figures S1H and S4J). The density from State II allowed us to build an additional model with a total of 20 bps of heteroduplex (Figure S4J). However, the additional 5 bp did not interact significantly with the protein and were exposed and flexible in solvent. This indicates that although the Fz1 ωRNA maturation process can produce a guide region up to 20-bp long, the lack of protection by the protein beyond 15 bp is limited, preventing the formation of a stable 20-bp heteroduplex.

### GtFz1 has a Non-canonical Catalytic Triad

**Figure 4.**
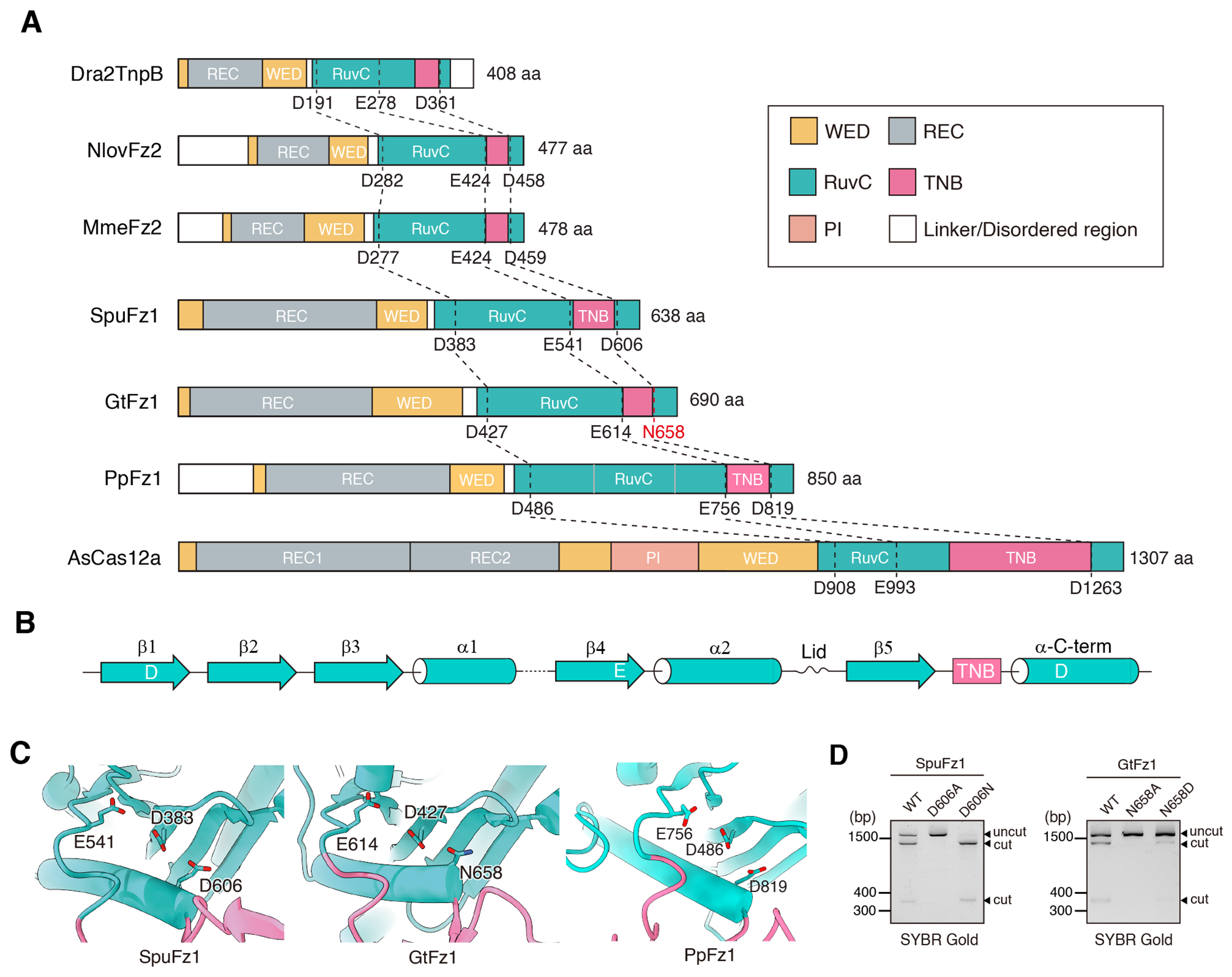
Catalytic triad of Fanzor1 and other RuvC nucleases. (**A**) Conserved catalytic motifs in the RuvC domain are shared among TnpB, Fz2, Fz1, and Cas12a. (**B**) Secondary structure of a canonical RuvC nuclease. (**C**) Structural details of the catalytic sites in SpuFz1, GtFz1 (which contains a non-canonical N in place of the canonical D in the third position), and PpFz1. (**D**) *In vitro* cleavage activity of SpuFz1 wild-type, D606A, D606N (left) and GtFz1 wild-type, N658A, and N658D (right).

Fz, TnpB, and Cas12a all use the same catalytic core in the RuvC domain to perform DNA double-strand cleavage (Figure 4A). In vitro cleavage experiments showed that GtFz1 operates at a similar optimal temperature as SpuFz1 for DNA cleavage (Figure S5A) ^2^. However, GtFz1 specifically requires Mg^2+^ ions to cleave DNA (Figure S5B), whereas SpuFz1 demonstrates a broader dependence on divalent cations for cleavage activity, functioning in the presence of Mg^2+^, Ca^2+^, Zn^2+^, and Mn^2+ 2^. In Fz, TnpB, and Cas12a, the RuvC core structure is primarily composed of five beta folds and one alpha helix. In this classic catalytic site pattern, the catalytic site consists of three residues: an aspartic acid (D) located on the second beta sheet, a glutamic acid (E) on the loop region between the fifth beta sheet and the TNB domain, and another D on the alpha helix (Figure 4B). In the structure of GtFz1, however, we found that the third catalytic residue of GtFz1 is not D but asparagine (N) (Figure 4C). Even though this substitution does not provide a positive charge, as is provided by the D residue, it still maintains the cleavage activity of GtFz1. Notably, introducing a comparable mutation into SpuFz1 (D606N) led to even higher cleavage activity compared to wildtype and the reverse mutation into GtFz1 (N658D) reduced its activity (Figure 4D). However, introducing the corresponding D-to-N mutations into Cas12 and TnpB do not increase their activities (Figure S5D). These results indicate that GtFz1 uses a non-canonical RuvC catalytic triad for DNA cleavage may be specifically beneficial for Fzs .

### Conformational Dynamics of GtFz1 in DNA Cleavage

In Cas12a, the recognition of the TAM sequence and local DNA strand separation allows hybridization with the guide segment of the ωRNA, thereby forming an R-loop structure ^21^. This process involves an RNA molecule infiltrating a dsDNA molecule, pairing with one strand while displacing the other, resulting in a three-stranded configuration. This R-loop creation is crucial for RNA-guided nucleases to precisely recognize and cleave target DNA ^22,23^.

**Figure 5.**
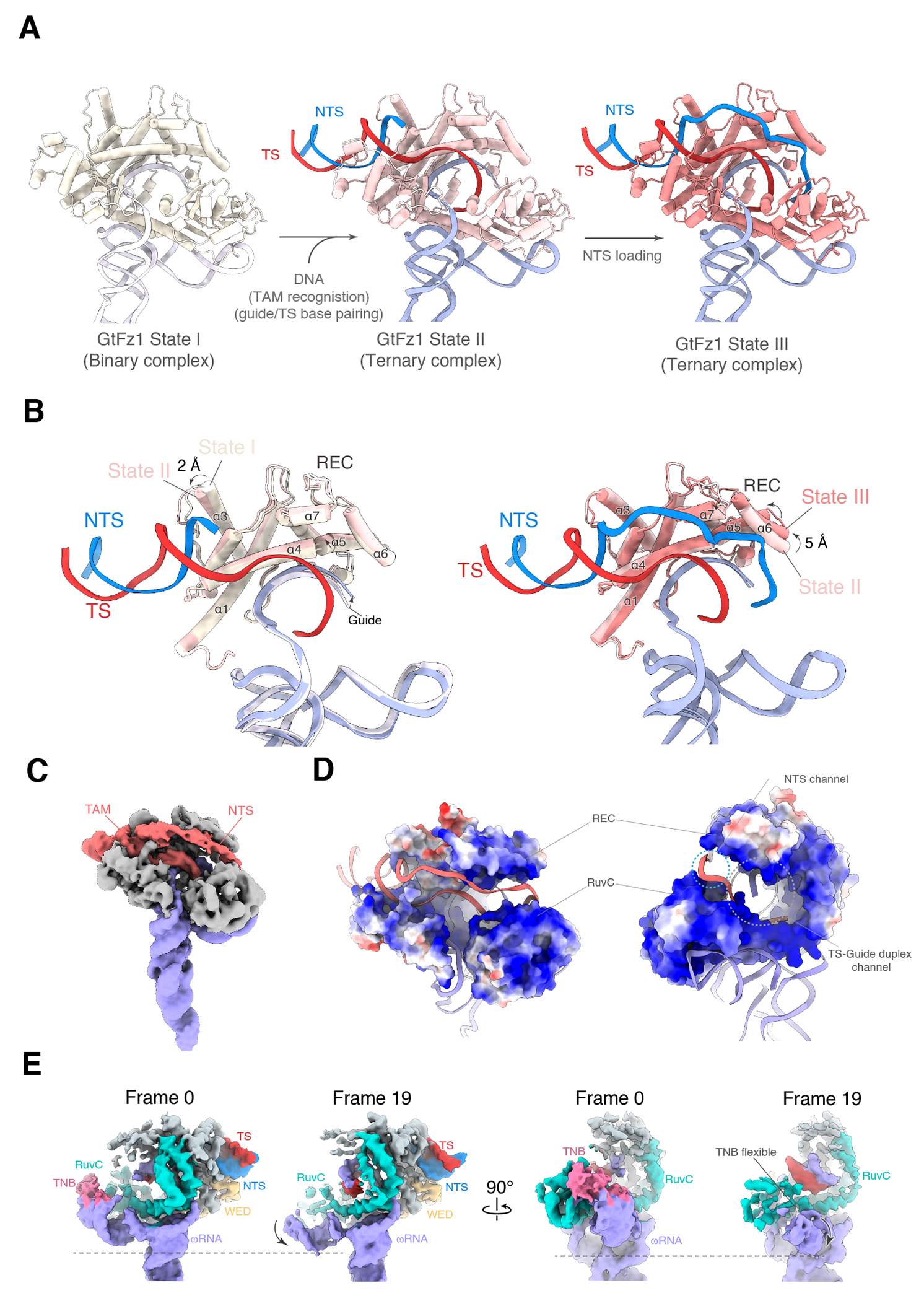
Structural features of GtFz1. (**A**) Structures of the GtFz1 State I (binary complex), State II (ternary complex), and State III (ternary complex). The target DNA of State II contains the TAM duplex and a single-stranded TS. The target DNA of State III is fully double-stranded. GtFz1 protein is colored tan in State I, pink in State II, and red in State III. (**B**) Comparison of the REC domain in GtFz1 State I with State II (top), and of State II with State III (bottom). (**C**) The EM map of the GtFz1 ternary complex displays the NTS loading into the RuvC domain. GtFz1 is colored gray, ωRNA is colored purple, and DNA is colored red. (**D**) Electrostatic potential mapping in GtFz1 illustrates the DNA binding channel. The green dashed circle indicates the channel for the DNA NTS binding. The white dashed circle indicates the channel for guide/TS heteroduplex binding. (**E**) 3D Variability Analysis (3DVA) reveals the conformational dynamics of SL1 and the RuvC/TNB domains. EM maps of the first frame and the last frame are shown from the same viewpoint to highlight the conformational change.

To elucidate the conformational changes before and after R-loop formation in Fz1, we compared the binary (State I) and ternary (State II and State III) complexes of GtFz1. Although the overarching structures of GtFz1 and its ωRNA are similar in these complexes (Figure 5A), there are clear conformational changes in the REC domain and the RNA guide region. Comparison of State I and State II, wherein the TAM has been recognized and the TS is forming base-pairs with the guide, shows that the α3 of the REC domain undergoes a shift of approximately 2 Å to accommodate TAM duplex binding. The guide region of the ωRNA, which is stabilized by both the WED and REC domains before and after DNA binding, shifts toward the REC domain by roughly 2 Å upon base pairing with the TS (Figure 5B). The unsharpened map of the GtFz1 State III reveals partial weak density of the NTS, spanning from the TAM duplex to the peripheries of the REC domain and RuvC (Figure 5C). These areas are characterized by positive surface charges, suggesting their role as interaction hubs for the NTS. Morphologically, GtFz1 exhibits a well-defined cleft structure essential for binding and stabilizing the RNA/DNA heteroduplex, with the cleft’s periphery allocated for binding the single-stranded NTS(Figure 5D). Comparison of GtFz1 State II and State III shows that the α6 of the REC domain undergoes a shift of approximately 5 Å upon the interactions with the NTS, suggesting that the REC domain plays a role in stabilizing the R-loop structure (Figure 5B).

We previously found that GtFz1 and SpuFz1 give rise to different patterns of cleavage products, with SpuFz1 consistently generating 5’ overhangs and GtFz1 generating more heterogeneous products ^2^. To investigate if this difference in the cleavage products arises from the flexibility of the active domain of GtFz1, we conducted a 3D Variability Analysis (3DVA). We found significant dynamics in SL1 of the ωRNA as well as the RuvC and TNB domains of GtFz1 (Figures 5E, Movie S1). This is in contrast to the stability observed in the corresponding region of SpuFz1 (Movie S2), supporting the finding that the cleavage site of GtFz1 is indeed flexible.

### SpuFz1 RuvC Domain Demonstrates Double-stranded DNA Binding Capabilities

To further explore the R-loop formation mechanisms in SpuFz1, we conducted *in vitro* cleavage assays and cryo-EM analysis using target DNA substrates (ds) with various phosphorothioate modifications on the TS and NTS. With these modified substrates, we observed that even when the cleavage site ^2^ and adjacent nucleotides on either both or one strand were blocked, cleavage of unblocked sites still occurred (Figures S6A–B). For the TS, cleavage was observed at the first unmodified nucleotide on the 5’ end, whereas for the NTS, cleavage occurred at the first unmodified nucleotide on both the 5’ and 3’ ends adjacent to the blocked segment (Figures S6A–B). This cleavage pattern correlates with the structure where the 3’ end of the TS is protected by duplex formation with the guide for 15 bps, whereas the NTS remains flexible.

**Figure 6.**
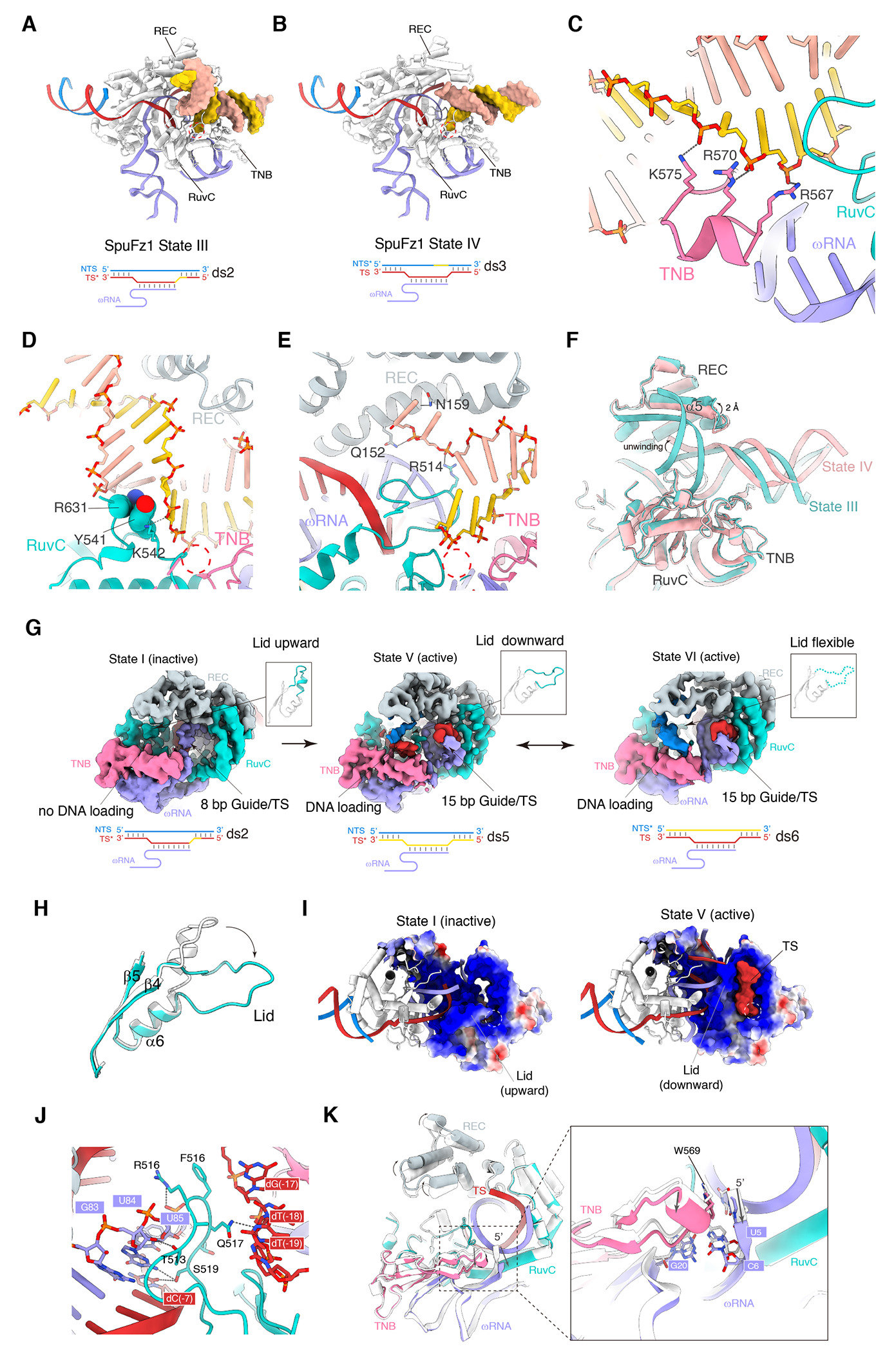
RuvC dsDNA loading and the lid regulation in SpuFz1 (**A**) Structure of SpuFz1 State III. dsDNA is bound to the large cleft formed by REC/RuvC/TNB domains. The dsDNA is bent by about 137° and the tip contacts the catalytic site of RuvC. SpuFz1 is colored white, ωRNA is colored purple, TS is colored red, NTS is colored blue. The dsDNA bound to RuvC is shown in surface and colored in gold and tan for each strand. The catalytic site in the RuvC domain is circled with a dashed line. A schematic diagram of the ternary complex formation is shown below. Substrate ds2, in which the TS is partially modified and NTS is unmodified, was used. (**B**) Structure of SpuFz1 State IV. dsDNA is bound to the large cleft formed by REC/RuvC/TNB domains in a distinct conformation from State III. SpuFz1 is colored white, ωRNA is colored purple, TS is colored red, NTS is colored blue. The dsDNA bound to RuvC is shown in surface and colored in gold and tan for each strand. The catalytic site in the RuvC domain is circled with a dashed red line. A schematic diagram of the ternary complex formation is shown below. Substrate ds3, where TS is unmodified and NTS is partially modified. (**C**) Close-up of charge interactions in the structure of SpuFz1 State III between residues of the TNB domain and dsDNA loaded onto the RuvC domain. (**D**) In the structure of SpuFz1 State III, residue R631 in the C-terminus loop of the RuvC domain, together with Y541, occupy the groove in the dsDNA that is bound to the RuvC domain. The catalytic site in the RuvC domain is circled with a dashed red line. (**E**) In the structure of SpuFz1 State IV, interactions formed by Q152, N159, and R514 stabilize the unwound DNA strand, which was not observed in State III. The catalytic site in the RuvC domain is circled with a dashed red line. (**F**) Structural alignment of SpuFz1 State III (green) and State IV (pink) showing that the dsDNA bound to the RuvC domain of these two states displays distinct conformations. The α5 of the REC domain is shifted by 2 Å. (**G**) The EM maps of SpuFz1 State I, State V, and State VI illustrate the conformational changes of the lid. In State I, an 8-bp guide/DNA duplex is formed. The lid is in an upward orientation, and no DNA is loaded onto the RuvC domain, representing an inactive state. In State V, a 15-bp guide/DNA duplex is formed. The lid is in a downward orientation, and the DNA TS is loaded onto the RuvC domain, representing an active state. In State VI, a 15-bp guide/DNA duplex is formed. The lid density is weak due to its structural flexibility. The DNA density is observed around the RuvC domain. Schematic diagrams of ternary complex formation are shown below (substrate ds2, TS is partially modified and NTS is unmodified; substrate ds5, TS is fully modified and NTS is unmodified; substrate ds6, TS is unmodified and NTS is fully modified). (**H**) Structural alignment of the lid of SpuFz1 in the inactive state (I) (white) with the active state (V) (cyan). The short helix of the lid in the active state is released. (**I**) Electrostatic potential mapping in SpuFz1 illustrates the structural changes from the inactive state (I) to the active state (V). The catalytic site is circled with a dashed line. The downward conformation of the lid forms a small cleft on the RuvC and TNB domain, allowing the DNA substrate to load onto the RuvC domain and approach the catalytic site. (**J**) Interactions of the lid in SpuFz1 State V. The lid is sandwiched by the guide/TS heteroduplex and the DNA segment loaded onto the RuvC domain. Hydrogen bonds are shown with dashed lines. (**K**) Structural alignment of SpuFz1 in the inactive state (I) with the active state (V). The entire complex in the inactive state is colored white. For the active state, the RuvC domain is colored cyan, ωRNA is colored purple, and the DNA TS is colored red. The inset shows the detailed conformational change at the 5’ end of the ωRNA, along with the TNB domain. This change is driven by the formation of the 15-bp guide/DNA heteroduplex.

To elucidate the structural basis for target cleavage of SpuFz1, we reconstituted the ternary complexes with these different 54 bp target DNA substrates (Figure S1H). Through cryo-EM single-particle analysis, we unexpectedly discovered density consistent with double-stranded DNA accommodated by the RuvC domain, when either the TS or the NTS were partially modified. (Figure 6A, B, and Figure S6C, D). In all states, the 15-bp guide/DNA heteroduplex was resolved. With partial modification to the TS, a 20-bp DNA duplex was observed anchored within the large cleft formed by the RuvC, TNB, and REC domains, which are rich in positively charged residues (Figure 6A, C, D, and Figure S6C). The DNA is bent by 137° around a putative scissile phosphate (or phosphorothioate), which is coordinated by two Mg^2+^ ions and the catalytic residues (D383, E541, and D606) of the RuvC domain. Non-continuous cryo-EM density between the putative scissile phosphate and the 3’ OH of the 5’ nucleotide suggests this may represent a post-cleavage state before product release. Of the 54 bp DNA substrate used for complex reconstitution, the cryoEM density unambiguously showed 12 bp for the TAM duplex and upstream bases and 15 nt of the TS within the guide/DNA heteroduplex. This leaves unassigned only 12 bp of further upstream DNA, and 15 bp downstream of the guide/DNA heteroduplex. Therefore, the >20 continuous basepairs visible for the RuvC-bound DNA likely correspond to a second DNA molecule.

Interestingly, in the structure with the partially modified NTS, the density for the strand nearest the RuvC active site terminates at the scissile phosphate, suggesting that the strand is cleaved and that the 5’ nucleotides are no longer stably bound (Figure 6B and S6D). The complementary strand shows that two nucleotides, initially base-paired with nucleotides near the catalytic site, are now disassociated and forming interactions with residues Q152 and N159 of the REC domain and R514 of the RuvC domain (Figure 6E). Correspondingly, the α5 of the REC domain shifts by approximately 2 Å (Figure 6F), indicating its flexibility to accommodate various dsDNA conformations and implying that the REC domain facilitates the unwinding of dsDNA bound to the RuvC domain. We name these states of SpuFz1 before and after nicked dsDNA product release and unwinding as State III (observed when the NTS was partially modified) and State IV (observed when the TS was partially modified), respectively. This evidence that dsDNA binds to the RuvC domain does not parallel findings in the structures of TnpB ^8,9^ and most of Cas12s ^11–16^, suggesting a distinct mechanism of R-loop formation in Fz1 relative to TnpB and Cas12.

### The RuvC Lid Regulates R-loop Structure and DNA Cleavage in SpuFz1

Given the capability of the SpuFz1 RuvC domain to cleave at positions other than the target site when the target site is chemically blocked, we performed additional cleavage assays with DNA substrates in which either the TS or NTS was fully modified (Figures S6E–F). These modifications included blocking both strands (ds4), only the TS (ds5), or only the NTS (ds6) (Figures S6E). The assays demonstrated that DNA could not be cleaved when both strands were modified, and cleavage was possible on one strand when its complementary strand was modified (Figure S6F). Further cryo-EM analysis yielded two distinct conformational maps for the SpuFz1-ds5 and SpuFz1-ds6 samples, which we classified as State V and State VI (Figure 6G and S1H). These states showed that, despite complete modifications preventing cleavage on either the TS or NTS, we could only observe partial DNA substrate density near the RuvC domain. This observation indicates a dynamic R-loop binding mode by SpuFz1.

To gain a more complete picture of the step-wise conformational changes of SpuFz1 cleavage, we examined the cryo-EM particles for variants with altered conformations. In the ds2 sample (TS partially modified), a small fraction (2.8% of particles with well-defined structural features from the SpuFz1-ds2 sample) exhibited only an 8-bp heteroduplex. Here, the “lid” region, a loop spanning residues 506-522 of the RuvC domain, adopts a short α helix in an upward orientation (Figure 6G). This helix interacts with another α helix (α1) of the RuvC domain. No clear density indicative of DNA binding to the catalytic site was observed. Thus, we classified this state as State I, as it is presumably the initial DNA-binding conformation.

We propose that state V may represent the next downstream conformation that we captured (Figure 6G). In state V, the lid forms a finger loop in the downward orientation, sandwiched by the guide/DNA duplex and the downstream DNA duplex (Figures 6G–J). The downward conformation of the lid forms interactions with the backbone of the dsDNA (Figure 6J). The lid releases the interactions with α1 of the RuvC domain, forming a small cleft between the lid and TNB domain. Thus, it appears that the formation of the 15-bp guide/DNA heteroduplex shifts the lid from an upward orientation to a downward orientation, creating space for the downstream target DNA to bind to the RuvC domain and reach the catalytic site (Figure 6I). To gain additional detail into the formation of this small cleft, we overlaid states I and V (Figure 6K) and examined the changes in the ωRNA. The visible terminal nucleotide of the 5’ end (U5) of the ωRNA is shifted by 6.5 Å in state V due to the formation of the 15-bp guide/DNA heteroduplex. The base group of U5 forms a stacked interaction with W569. Meanwhile, the bp of the second visible terminal nucleotide, C6 and G20 at the SL1 of the ωRNA, together with the short helix of the TNB domain, were shifted by about 1 Å (Figure 6K). These movements together with the flipped orientation of the lid, create the small cleft that stabilizes the DNA at the catalytic site. The comparison also shows that the REC domain accommodates downstream DNA binding by shifting the structure by about 3 Å, with charged interactions formed between the REC domain and the backbone of the DNA. In state VI, the density of the lid appears exceptionally weak (Figure 6G), indicating a diminished role in facilitating NTS single-strand loading. These states emphasize the subtle yet significant roles of the lid in modulating DNA binding and cleavage. To further validate the importance of the lid, we performed *in vitro* cleavage assays with truncations and point mutations on the lid of RuvC, showing that the lid is crucial for SpuFz1 activity (Figure S7A).

### The Dynamics of ωRNA 5’ Regulation in Heteroduplex-Driven Activation in PpFz1

**Figure 7.**
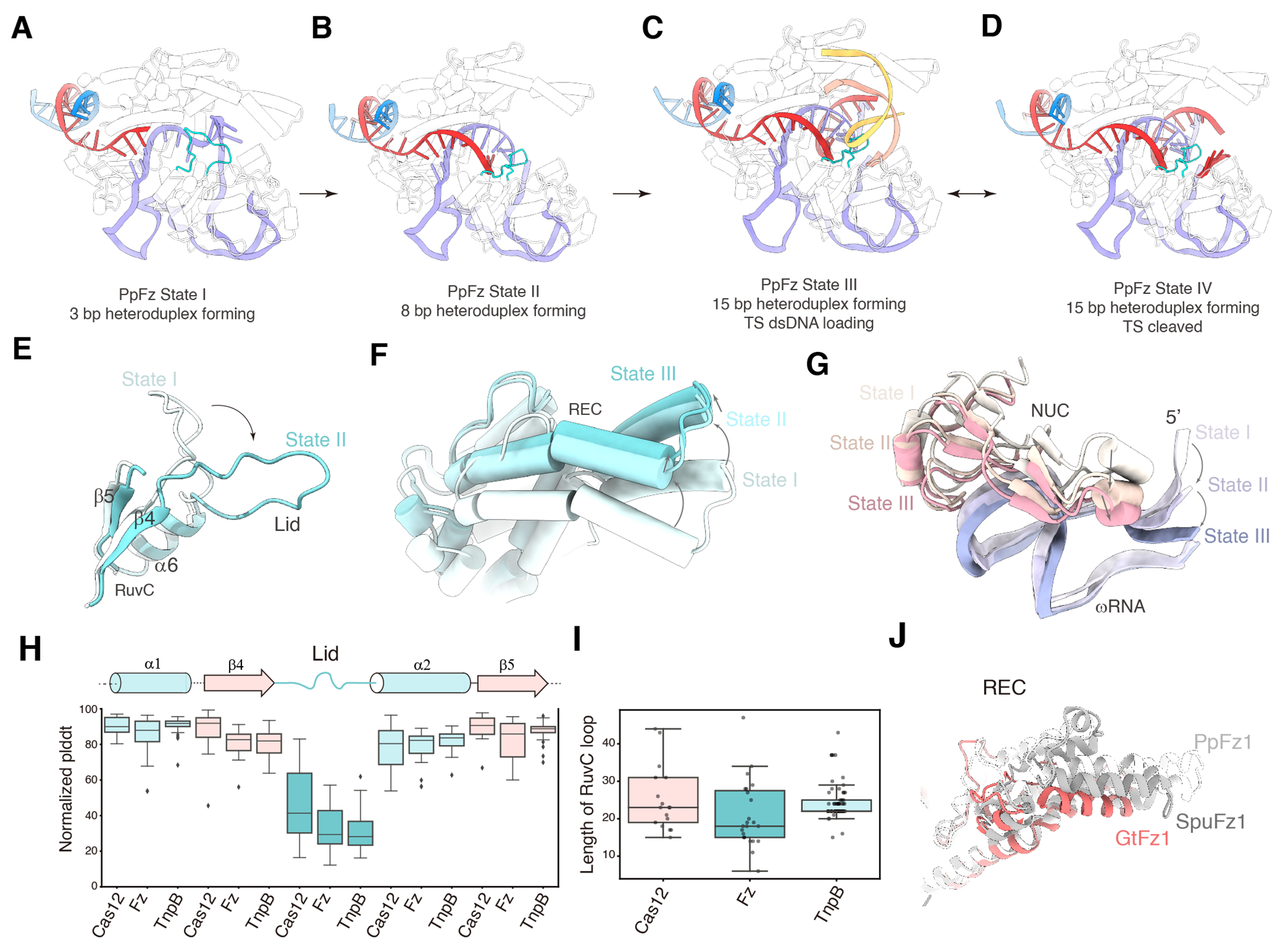
PpFz1 DNA loading and cleavage mechanisms (**A-D**) Structures illustrating the activation stages of PpFz1, detailing the conformational changes, DNA loading, and cleavage processes. Protein domains, ωRNA, and target DNA are colored as in Figures 6. (**E**) Structural alignment of the lid of PpFz1 comparing the inactive state (I) (white) with the intermediate state (II) (cyan). (**F**) Structural alignment of the REC domain comparing the inactive state (I), intermediate state (II), and active state (III). (**G**) Structural alignment at the 5’ end of the ωRNA and the TNB domain comparing the inactive state (I), intermediate state (II), and active state (III). (**H**) Comparison of the Predicted Local Distance Difference Test (pLDDT) for the lid region and its surrounding secondary structures (upstream: α1, β2; downstream: α2, β5) across 59 representatives of Cas12, Fzs, and TnpB. pLDDT scores were normalized as described in Methods, and the scores for each region of each protein are provided and detailed in Data S1. (**I**) Comparison of the length distribution (in residues) of the lid regions across 59 representatives of Cas12s, Fzs, and TnpBs. The lengths for each protein are provided in Data S1. (**J**) Structural alignment of the REC domain comparing the active states of GtFz1 (red), SpuFz1 (gray), and PpFz1 (white).

Multiple conformational states of PpFz1 were also obtained; four states were identified, with the guide/DNA heteroduplex 3-bp long in State I, 8-bp long in State II, 15-bp long in both State III and State IV (Figures 7A–D). In State I, the lid of PpFz1 remains in an upward orientation, and no DNA is bound to the RuvC domain. The ωRNA guide segment (C50-C58) encircles the lid and contacts the REC domain (Figure 7A), which differs from the guide conformation observed in GtFz1 in its inactive state (binary complex) (Figures 5A–B). In State II, the lid shifts to a downward orientation due to the formation of an 8-bp guide/DNA duplex (Figure 7E), although there is still no distinct density of DNA binding to the RuvC domain. This suggests that this 8-bp state represents an intermediate, which may not be favorable for DNA loading and cleavage (Figure 7B). In State III, a 15-bp guide/DNA heteroduplex is formed, and a dsDNA duplex is loaded onto the RuvC domain (Figure 7C), indicating an active state. In State IV, the loaded DNA is cleaved, and a ssDNA product binds to the small cleft formed by the RuvC lid and TNB domain, with the end nucleotide contacting the catalytic site (Figure 7C).

Structural comparisons of the inactive state (I), intermediate state (II), and active state (III) reveal that the REC domain shifts to widen the large cleft by 8 Å from State I to State II, and by an additional 2 Å from state II to state III (Figure 7F). The 5’ nucleotide A5 and the short helix of the TNB domain are pushed outward by the lid’s conformational change and by the formation of the guide/DNA duplex, moving 5.7 Å between State I and State II, and an additional 2.3 Å between State II and State III (Figure 7G). The conformational changes in the RuvC/TNB domains create a small cleft, in which the DNA substrate is stabilized and gains access to the catalytic site. Concurrently, the outward shift of the REC domain enables the downstream dsDNA to be stabilized within the large cleft of PpFz1. These structures capture the process of target DNA cleavage and illuminate how the large and small clefts formed by the RuvC, REC, and TNB domains, along with the lid, regulate DNA binding, unzipping, and cutting. The diverse structural states demonstrate the critical role of the lid region of the RuvC domain in DNA binding and strand switching.

Previous research on Cas12s and TnpB has revealed the lid region of these proteins is structurally dynamic ^8,11^. Our current study illustrates how the lid region influences the activation of Fz1 by examining structures in various states. To delve deeper into the role of the lid in Cas12 family proteins, we analyzed the predicted Local Distance Difference Test (pLDDT) scores and lid lengths across various Cas12, Fz, and TnpB AlphaFold (AF) models (see Methods). The findings show that, irrespective of the protein, the lid region consistently exhibits lower pLDDT values compared to the adjacent alpha-helices and beta-sheets (Figure 7H), suggesting a propensity for multiple conformations. The predicted lid lengths for these three protein families are notably similar, with median lengths ranging from 20 to 22 amino acids (Figure 7I). In the available Cas12 protein structures, the lid regions of the RuvC domain are strategically situated between the guide/DNA heteroduplex and the catalytic site, implying that a common mechanism may underlie protein activation across these families. The utilization of the lid as a regulatory “switch” could represent a conserved evolutionary feature, possibly derived from TnpB.

## DISCUSSION

The structures of the three Fz1 complexes are largely similar, sharing a common protein domain architecture and shared folding patterns in key ωRNA regions. This uniformity underlies their analogous mechanisms for DNA recognition and cleavage. However, there are distinct structural features among these three proteins, highlighting the diversity within the Fz protein family. A unique aspect of GtFz1 is the presence of asparagine (N) in the catalytic triad, contrasting with the canonical aspartic acid (D) at the same position in SpuFz1, mutation of which to N enhances *in vitro* activity and specific for Fzs. The alternative N residue in the catalytic triad may enhance enzymatic activity by influencing cation coordination. This observation provides insight into the catalytic dynamics of the RuvC domain and suggests strategies for engineering Fzs to enhance their activity.

The Fanzor RuvC Insertion (FRI) domain in PpFz1 is another distinct feature. We found that this domain mediates binding to a cyclophilin (Figure S2E), which may be important for the physiological function of Fz in the native host organism. Our data indicate that PpFz can bind Cyp from yeast and other organisms. As a mycoparasites, *Parasitella parasitica* may use Fz to interact with other species. We only found the FRI domain in *Parasitella parasitica* and *Rhizopus*, a genus of the Mucorales order. Interestingly, *Parasitella* are mycoparasites of other fungi, including those of the Mucorales order ^24^. The presence of the FRI domain in these organisms suggests Fz1 may play a role in this parasitic relationship, possibly by facilitating transposon-mediated gene transfer. The structure of the PpFz1-cyclophilin complex hints at the significant roles cyclophilins may play in regulating Fz function across various biological processes, such as splicing, meiosis, plant pathology, pathogen infection, and sporulation ^10,25,26^.

Although both Fz and Cas12 evolved from the TnpB family ^6^, our structural analyses delineate clear distinctions in the DNA recognition and cleavage mechanisms among these families. In contrast to Cas12, Fz1 proteins utilize a loop insertion strategy to bridge the gap between the TAM and guide sequences, initiating DNA unwinding. This contrasts with the strategies employed by Cas12 and TnpB proteins, where Cas12a uses a short helix-loop-helix (HLH) for DNA unwinding ^27^, and TnpB relies on the intrinsic propensity of DNA to unwind spontaneously ^8^. Moreover, our structures elucidate distinct capabilities of Fz1 in stabilizing R-loop structures. Unlike the more expansive REC domain in Cas12a, which stabilizes a roughly 20-bp heteroduplex ^11^, the smaller REC domain in Fz1 can stabilize at most a 15-bp heteroduplex. The REC domain is smaller yet in TnpB. The additional REC domain structure in Fz1 does not significantly contribute to heteroduplex stabilization but rather to the R-loop stabilization of target DNA loaded onto the RuvC domain. This dynamic shift from an inactive to active state in the REC domain is crucial for stabilizing the single-stranded NTS in GtFz1 and the double-stranded DNA in SpuFz1 and PpFz1. This adaptation of the DNA unwinding mechanism and R-loop regulation by the REC domain may be crucial for the function of Fz in the complex chromatin environment of eukaryotic cells, representing an evolutionary innovation in these systems. The intricate organization of eukaryotic chromatin requires precise mechanisms to access and manipulate DNA, a challenge that Fz proteins meet through their specialized structural features. Together, these insights advance our understanding of the mechanisms used by RNA-guided endonucleases and provide a solid foundation for engineering Fanzor for gene editing technologies.

### Limitations of the study

Despite revealing the various conformations of Fz1 structures and their role in regulating DNA cleavage during R-loop formation, the captured structures do not fully represent the complete R-loop. Additionally, the local resolution near the RuvC domain for the DNA substrate is relatively low, making it challenging to determine how Fz dynamically adapts to different cleavage positions. These limitations are due to the inherent dynamic nature of R-loop formation and the DNA cleavage process. Future studies with higher resolution and real-time observations are necessary to fully elucidate these mechanisms. Other open questions remain about the possible additional biological functions of the large ωRNA used by GtFz1 and the association between PpFz1 and Cyp. Future studies focused on these aspects may reveal key insight about the biological function of Fzs.

## STAR★Methods

### Resource availability

#### Lead contact

Further information and requests for resources and reagents should be directed to and will be fulfilled by the Lead Contact, Feng Zhang (zhang@broadinstitute.org )

#### Materials availability

Plasmids generated in this study are available from Addgene or by request. All other reagents are available upon request.

#### Data and code availability

- The atomic coordinates have been deposited in the Protein Data Bank. The EM map has been deposited in the Electron Microscopy Data Bank (Table S1).
- The script used for the unbiased matching of the yeast proteome to the target electron microscopy density map is available in the supplementary materials.
- Any additional information required to reanalyze the data reported in this paper is available from the lead contact upon request.

### Experimental model and study participant details

#### Cell culture

*S. cerevisiae* BCY123 strain (a kind gift from the K. Nagai laboratory, MRC Laboratory of Molecular Biology, Cambridge) was cultured in YM4 LMB media (0.67% yeast nitrogen base without amino acids, 0.5% casamino acids, 0.002% adenine, and 0.002% tryptophan) supplemented with 2% raffinose for growth at 30°C with shaking at 180 rpm until an optical density at a wavelength of 600 nm of 1.0 was reached. Protein expression was then induced in the presence of 2% galactose.

### Method details

#### Cloning

Plasmids used in this study were cloned using general cloning methodologies including Gibson assembly with NEBuilder HiFi DNA Assembly Master Mix (New England Biolabs, E2621L) and KLD Enzyme Mix (New England Biolabs, M0554S). The Stbl3 *E. coli* strain (Thermo Fisher, C737303) was used for DNA cloning. Fanzor1 (Fz1) ORF and predicted 3′ inverted repeat (IR) regions were cloned under a GAL-GAPDH hybrid promoter in pRS426-URA3. A 10xHis-maltose-binding protein (MBP) tag was inserted between the start and second codons of Fz1. The sequences of cloned constructs were confirmed by whole-plasmid sequencing following Tn5 tagmentation after mini-prep of plasmids^28^ with QIAprep reagents (QIAGEN, 27106).

#### Fz1 RNP expression and purification

Fz1 orthologs were expressed in *S. cerevisiae* and MBP-affinity purified. The expression vector was transformed into a yeast BCY123 strain ^29^ and selected on SD-URA plates. Colonies on half of the petri dish were scraped and transferred into a 50-mL starter culture of YM4 LMB media supplemented with 2% raffinose for 17 hours, which was used to inoculate 1 L of YM4 LMB media supplemented with 2% raffinose and 100 μg/ml ampicillin for growth at 30°C and shaking at 180 rpm until an OD600 of 1.0 was reached. Protein expression was induced in the presence of 2% galactose for 16 hours. The cells were harvested by centrifugation for 10 minutes at 4°C at 4000 rpm (Beckman Coulter Avanti J-E, rotor JLA8.100). The cell pellet was resuspended in 500 ml of MQ water to remove residual media, and pelleted again by centrifugation for 10 minutes at 4°C at 4000 rpm. The pellet was kept frozen at −80°C for further usage.

All purification steps were performed at 4°C. The yeast cell pellet was resuspended in a buffer containing 20 mM HEPES pH 7.5, 150 mM NaCl, 2 mM MgCl_2_, and 4.5 mM TCEP supplemented with EDTA-Free Protease Inhibitor Cocktail (MedChem Express HY-K0010). The cell suspension was then added dropwise into liquid nitrogen in an ice bucket, and the resulting frozen beads were ground with a few pellets of dry ice in a pre-chilled coffee grinder (CG-618-SHARDOR). The frozen yeast powder was thawed and cleared by centrifugation for 35 minutes at 4°C at 15000 rpm (Beckman Coulter Avanti J-E, rotor JLA-16.25). The cleared lysate was applied to 3 mL of packed Amylose Resin (NEB) for 3 hours, followed by washing with 100 mL of buffer containing 20 mM HEPES pH 7.5, 150 mM NaCl, 2 mM MgCl_2_, and 4.5 mM TCEP. The MBP-tagged Fz-ωRNA RNPs were then eluted with the same buffer supplemented with 10 mM Maltose (Sigma-Aldrich, M9171) and concentrated using an Amicon Ultra-15 Centrifugal Filter Unit (50KDa NMWL, Millipore UFC905024). The resulting elution was tested for the presence of the protein by NuPAGE (Invitrogen) and eStain L1 Protein Staining System (GenScript). For testing the presence of RNA, 1 μl Proteinase K (NEB) was mixed with 15 μl eluted RNP for 15 minutes and evaluated by 15% TBE-Urea polyacrylamide gels (Thermo Fisher Scientific).

#### Fanzor RNP TAM screen

Purified Fz1 RNP and 25 ng of TAM library plasmid were supplemented with MgCl_2_, and the 10 μL reaction mixture (10 mM Tris-HCl, 50 mM NaCl, 5 mM MgCl_2_, 2 mM 2-Mercaptoethanol and 1% glycerol, pH 8.0) was incubated at 37°C for 4 hours, then quenched by adding 10 μg RNase A (Qiagen) and 8 units Proteinase K (NEB) each followed by a 15-minute incubation at room temperature. DNA was extracted by PCR purification and adaptors were ligated using an NEBNext Ultra II DNA Library Prep Kit for Illumina (NEB) using the NEBNext Adaptor for Illumina (NEB). Following adaptor ligation, cleaved products were amplified specifically using one primer specific to the TAM library backbone and one primer specific to the NEBNext adaptor with a 12-cycle PCR using NEBNext High Fidelity 2X PCR Master Mix (NEB) with an annealing temperature of 63°C, followed by a second 18-cycle round of PCR to further add the Illumina i5 adaptor. Amplified libraries were gel extracted and subject to single-end sequencing on an Illumina MiSeq with Read1 80 cycles, Index1 8 cycles and Index2 8 cycles. TAMs were extracted, and an enrichment score for each TAM was calculated by filtering for all TAMs present more than once and normalizing to the TAM frequency in the input library. A position weight matrix based on the enrichment score was generated, and Weblogos ^31^ (https://weblogo.berkeley.edu/logo.cgi) were visualized based on this position weight matrix using a previously published custom script ^30^. A 5’-ATN-3’ TAM for PpFz1 was confirmed (Figure S4C).

#### In vitro cleavage assays

Double-stranded DNA (dsDNA) substrates were produced by PCR amplification of plasmids or synthesized DNA fragments containing the target sites and the TAM sequences. Target cleavage assays were performed in a 10 μl reaction mixture containing 50 ng of substrate, 2 μg of protein in a final 1x reaction buffer of 20 mM HEPES pH 7.5, 150 mM NaCl, and 5 mM MgCl_2_. Assays were allowed to proceed at 37°C for 1 hour. Reactions were then treated with RNase A (Qiagen) and Proteinase K (NEB) and purified using a PCR cleanup kit (Qiagen). For screening metal ions, MgCl_2_ was eliminated from the reaction buffer through Amicon Ultra-0.5 Centrifugal Filter Unit, and the indicated metal was added. Purified DNA substrates after the assays were resolved by gel electrophoresis on E-gel 2% (dsDNA substrates), 15% TBE-Urea polyacrylamide gels (Thermo Fisher Scientific).

#### Preparation of the Fz1-ωRNA-target DNA ternary complexes

For GtFz1-ωRNA-target DNA ternary complex, two samples were prepared with different guide sequences (Sample 1: native guide, Sample 2: PSP1 guide). The purified GtFz1 RNP1 with native guide and GtFz1 RNP2 with PSP1 guide were loaded on a Superose 6 Increase 10/300 column (Cytiva) equilibrated with a buffer containing 20 mM HEPES pH 7.5, 150 mM NaCl, 2 mM MgCl_2_, and 4.5 mM TCEP. The fractions of RNP complexes were pooled and concentrated to 3 mg/ml using Amicon Ultra-15 Centrifugal Filter Unit (50KDa NMWL, Millipore UFC905024) for ternary complex formation. For ternary complex reconstitution of sample 1, the GtFz RNP1 (native guide) was mixed with a 48-nt DNA target strand (GtFz_ntv_TS) and an 18-nt non-target strand (GtFz_ntv_NTS) at a molar ratio of 1:2:2 and incubated at 37°C for 30 min. For ternary complex reconstitution of sample 2, the GtFz1 RNP2 (PSP1 guide) was mixed with an 81-nt DNA target strand (GtFz_PSP1_TS) and an 81-nt non-target strand (GtFz_PSP1_NTS) at a molar ratio of 1:2:2 and incubated at 37°C for 1 hour. The mixture of GtFz1 ternary complexes was applied onto glow-discharged UltrAuFoil R 1.2/1.3, 300 mesh, Gold (Quantifoil). The grids were blotted for 3 seconds under 100% humidity at 4°C and then vitrified by plunging into liquid ethane using a Vitrobot Mark IV (Thermo Fisher Scientific).

For SpuFz1-ωRNA-target DNA ternary complexes, the SpuFz1 RNP was concentrated using an Amicon Ultra-15 Centrifugal Filter Unit (50KDa NMWL, Millipore UFC905024) to 5 mg/ml and mixed with 4 different dsDNA substrates, resulting in 4 ternary complex samples.The 4 different dsDNA substrates share the same TAM and guide sequence but had different lengths and phosphorothioate modifications. For SpuFz1-ds2 complex, the DNA target stand was partially modified and the non-target strand was unmodified. For SpuFz1-ds3 complex, the DNA where target stand was unmodified and the non-target strand was partially modified. For SpuFz1-ds5, the DNA target stand was fully modified and the non-target strand was unmodified. For SpuFz1-ds6, the DNA target stand was unmodified and the non-target strand was fully modified. The dsDNA substrates were annealed and mixed with SpuFz1 RNP at 37°C for 1 hour. The mixture of SpuFz1 ternary complexes was applied onto glow-discharged CryoMatrix^®^ R1.2/1.3 300-mesh gold holey grids with amorphous alloy film (Zhenjiang Lehua Technology Co., Ltd). The grids were blotted for 3 seconds under 100% humidity at 4°C and then vitrified by plunging into liquid ethane using a Vitrobot Mark IV (Thermo Fisher Scientific).

For PpFz1-ωRNA-target DNA ternary complexes, purified RNP was mixed with TEV protease at 4°C for 6 hours. The mixture was incubated with 1 ml TALON resin (Takara Clontech) at 4°C for 3 h in a buffer containing 20 mM HEPES, pH 7.5, 150 mM NaCl, 5 mM MgCl_2_, 4.5 mM TCEP, and 15 mM Imidazole. The resin was washed with 20 mL of a buffer containing 20 mM HEPES, pH 7.5, 150 mM NaCl, 5 mM MgCl_2_, 4.5 mM TCEP, and 20 mM Imidazole. The flow-though was collected and concentrated using an Amicon Ultra-15 Centrifugal Filter Unit (50KDa NMWL, Millipore UFC905024) to 500 ul. The sample was mixed with a 57-nt DNA target stand (PpFz_TS) and a complementary 57-nt non-target strand (PpFz_NTS) at 37°C for 1 hour. The mixture of PpFz1 ternary complexes was applied onto glow-discharged CryoMatrix^®^ R1.2/1.3 300-mesh gold holey grids with amorphous alloy film (Zhenjiang Lehua Technology Co., Ltd). The grids were blotted for 3 seconds under 100% humidity at 4°C and then vitrified by plunging into liquid ethane using a Vitrobot Mark IV (Thermo Fisher Scientific).

#### Cryo-EM data collection

For the GtFz1-ωRNA-target DNA ternary complex sample 1, the prepared grids were transferred to Thermo Scientific Titan Krios G3i cryo TEM using a K3 direct detector (Gatan) operated in super-resolution mode with 2-fold binning, and an energy filter with slit width of 20 eV. Micrographs were collected automatically using EPU in AFIS mode, yielding 12,579 movies at 130,000x magnification with a real pixel size of 0.663 Å, with defocus ranging from −0.8 μm to −2.2 μm with an exposure time of 1.01 second, fractionated into 40 frames and a flux of 25.8 e−/pix/s giving a total fluence per micrograph of 59.28 e−/Å2. For the GtFz1-ωRNA-target DNA ternary complex sample 2, the prepared grids were transferred to the EF-Krios (Thermo Fisher Scientific) operating at 300 kV with a GatanK3 imaging system collecting at 105,000x nominal magnification. The calibrated pixel size of 0.4125 Å was used for processing. Movies were collected using Leginon 3.6^30^ at a dose rate of 32.32 e−/Å2/s with a total exposure of 1.80 seconds, for an accumulated dose of 58.18 e−/Å2. Intermediate frames were recorded every 0.03 seconds for a total of 60 frames per micrograph. A total of 6,284 images were collected at a nominal defocus range of 0.5 – 2.5 μm.

For the SpuFz1-ωRNA-target DNA ternary complex samples, the prepared grids were transferred to the EF-Krios (Thermo Fisher Scientific) operating at 300 kV with a GatanK3 imaging system collecting at 105,000x nominal magnification. The calibrated pixel size of 0.4125 Å was used for processing. Movies were collected using Leginon 3.6^30^. Data were collected at a dose rate of 29.17 e−/Å2/s for SpuFz1-ds2 complex, 28.33 e−/Å2/s for SpuFz1-ds3 complex, 26.67 e−/Å2/s for SpuFz1-ds5 complex, and 26.67 e−/Å2/s for SpuFz1-ds6 complex with a total exposure of 1.80 seconds, resulting an accumulated dose of 52.51 e−/Å2 for SpuFz1-ds2 complex, 51.0 e−/Å2 for SpuFz1-ds3 complex, 48.0 e−/Å2 for SpuFz1-ds5 complex, and 48.0 e−/Å2 for SpuFz1-ds6 complex. Intermediate frames were recorded every 0.03 seconds for a total of 60 frames per micrograph. A total of 19,873 images (SpuFz1-ds2), 3,070 images (SpuFz1-ds3), 5,628 images (SpuFz1-ds5), and 6,464 images (SpuFz1-ds6) were collected at a nominal defocus range of 0.5 – 2.5 μm.

For the PpFz1-ωRNA-target DNA ternary complex, the prepared grids were transferred to the EF-Krios (Thermo Fisher Scientific) operating at 300 kV with a GatanK3 imaging system collecting at 105,000x nominal magnification. The calibrated pixel size of 0.4125 Å was used for processing. Movies were collected using Leginon 3.6 ^31^ at a dose rate of 26.92 e−/Å2/s with a total exposure of 1.80 seconds, for an accumulated dose of 48.47 e−/Å2. Intermediate frames were recorded every 0.03 seconds for a total of 60 frames per micrograph. A total of 7,678 images were collected at a nominal defocus range of 0.5 – 2.5 μm.

#### Cryo-EM data processing

Image processing was performed on CryoSPARC v4.2.0 ^32^ and RELION 4.0 ^33^. Image stacks were subjected to beam-induced motion correction using MotionCor2.0 ^34^. Contrast transfer function (CTF) parameters for each non-dose-weighted micrograph were determined by CTFFIND4 ^37^. On-the-fly particle picking was done by Warp ^36^.

For GtFz1 Sample 1, automated particle picking yielded 1,279,545 particles, which were extracted on a binned dataset with a pixel size of 1.326 Å and were subjected to reference-free 2D classification and 7 rounds of heterogeneous refinement, producing 468,892 particles with well-defined structural features of a ternary complex. These particles were re-extracted with a pixel size of 0.663 Å and subjected to non-uniform refinement^36^, which generated a map with an indicated global resolution of 3.28 Å at a Fourier shell correlation (FSC) of 0.143. We defined this map as GtFz1 state II. The particles were subjected to 3D classification, a subset with 10,507 particles showing binary complex features. These particles were then subjected to non-uniform refinement, generating a map with an indicated global resolution of 4.70 Å at a FSC of 0.143. We defined this map as GtFz1 state I.

For GtFz1 Sample 2, automated particle picking yielded 1,455,905 particles, which were extracted on a binned dataset with a pixel size of 2.475 Å and were subjected to reference-free 2D classification and 8 rounds of heterogeneous refinement, producing 328,609 particles with well-defined structural features of a ternary complex. These particles were re-extracted with a pixel size of 0.825 Å and subjected to non-uniform refinement^37^, which generated a map with an indicated global resolution of 3.00 Å at a Fourier shell correlation (FSC) of 0.143. We defined this map as GtFz1 state III. The unsharpened map shows DNA non-target strand bound to the REC and RuvC domains compared to the ternary complex of GtFz1 sample 1. DeepEMhancer ^38^ was used for generating the sharpened map.

For SpuFz1-ds2 complex, automated particle selection yielded 7,851,258 particles, which were extracted on a binned dataset with a pixel size of 1.65 Å and were subjected to reference-free 2D classification and 5 rounds of heterogeneous refinement, producing 1,729,361 particles with well-defined structural features. These particles were re-extracted with a pixel size of 0.825 Å and were subsequently subjected to an additional 2 rounds of heterogeneous refinement and 1 round of 3D classification. A subset with 201,468 particles was subjected to an additional round of 3D classification, resulting in a subset with 47,576 particles that showed an 8-bp heteroduplex formed. This was then subjected to non-uniform refinement ^37^, which generated a map with an indicated global resolution of 3.29 Å at a Fourier shell correlation (FSC) of 0.143. We defined this map as SpuFz1 state I. DeepEMhancer ^38^ was used to generate the sharpened map. Another subset with 171,210 particles was subjected to an additional round of 3D classification, resulting in a subset with 31,906 particles that showed 20-bp heteroduplex density. This subset was then subjected to non-uniform refinement, which generated a map with an indicated global resolution of 3.26 Å at a Fourier shell correlation (FSC) of 0.143. We defined this map as SpuFz1 state II. The unsharpened map of state II was used to visualize the extended heteroduplex density. Another subset with 193,966 particles showing clear structural features of dsDNA loaded onto the RuvC domain was subjected non-uniform refinement, which generated a map with an indicated global resolution of 2.88 Å at a Fourier shell correlation (FSC) of 0.143. We defined this map as SpuFz1 state III. DeepEMhancer was used to generate the sharpened map.

For SpuFz1-ds3, automated particle picking yielded 1,243,815 particles, which were extracted with a pixel size of 0.825 Å and were subjected to reference-free 2D classification and 6 rounds of heterogeneous refinement and 3D classification, producing 129,301 particles with well-defined structural features. These particles were subsequently subjected to 3D classification and non-uniform refinement, producing a class with 34,628 particles showing better density, which generated a map with an indicated global resolution of 3.27 Å at a Fourier shell correlation (FSC) of 0.143. We defined this map as SpuFz1 state IV.

For SpuFz1-ds5 sample, automated particle picking yielded 1,240,493 particles, which were extracted on a binned dataset with a pixel size of 2.475 Å and were subjected to reference-free 2D classification and heterogeneous refinement, producing 558,533 particles with well-defined structural features. These particles were re-extracted with a pixel size of 0.825 Å and were subsequently subjected to additional heterogeneous refinement for improving the orientation issue. A subset with 245,331 was subjected to non-uniform refinement, which generated a map with an indicated global resolution of 3.22 Å at a Fourier shell correlation (FSC) of 0.143. We defined this map as SpuFz1 state V.

For SpuFz1-ds6 sample, automated particle picking by Warp yielded 851,391 particles, which were extracted with a pixel size of 0.825 Å and were subjected to reference-free 2D classification and 3 rounds of heterogeneous refinement, producing 227,222 particles with well-defined structural features. These particles were subsequently subjected to non-uniform refinement, which generated a map with an indicated global resolution of 3.41 Å at a Fourier shell correlation (FSC) of 0.143. This map we defined as SpuFz1 state VI. DeepEMhancer was used to generate the sharpened map.

For the PpFz1-ωRNA-target DNA ternary complex, automated particle selection yielded 3,535,752 particles, which were extracted with a pixel size of 0.825 Å and were subjected to reference-free 2D classification, Ab-initial reconstruction, and 9 rounds of heterogeneous refinement, producing 88,338 particles with well-defined structural features. These particles were subsequently subjected to 3D classification, with 4 maps with distinct conformations, which we defined as PpFz1 state I to state IV. For each one, non-uniform refinement was conducted, resulting in a map of state I with 15,797 particles with an indicated global resolution of 3.47 Å, a map of state II with 14,604 particles with an indicated global resolution of 3.52 Å, a map of state III with 18,003 particles with an indicated global resolution of 3.20 Å, and a map of state IV with 16,430 particles with an indicated global resolution of 3.15 Å.

#### Model building

For the structures of the GtFz1 complexes and PpFz1 complexes, protein models predicted by AlphaFold2 ^42,44^ and an ωRNA model generated by RNAcomposer ^40^ were used as initial models. For SpuFz1 complexes, the model of the SpuFz1-ωRNA-target DNA ternary complex (PDB: 8GKH) was used as the initial model. For the complexes with DNA substrates loaded onto the RuvC domain and undetermined DNA sequences, complementary poly A and poly T DNA sequences were used for model building for analysis.The models were docked into the cryo-EM density maps using ChimeraX 1.7 ^41^, followed by iterative manual adjustment and rebuilding in ISOLDE ^42^ and Coot 0.8.9^43^, against the cryo-EM electron density maps. Real space and reciprocal refinements were performed using PHENIX 1.18^45^. The model statistics were validated using MolProbity 4.5 ^46^. Structural figures were prepared in ChimeraX 1.7, and PyMOL (https://pymol.org/2/). The final refinement statistics are provided in Table S1.

#### LC-MS/MS

Digestions were performed with S-trap micro spin columns from Protifi per the manufacturer’s protocol. Volumes in the protocol were adjusted for samples volume, 10 mM DTT(final concentration) was used instead of TCEP and 20 mM iodoacetamide(final concentration) was used instead of MMTS. After adding the DTT, sample tubes were placed on a heating block for 10 minutes at 95°C. Proteins were then alkylated with iodoacetamide, after adding the iodoacetamide samples incubated at RT for 30 minutes in the dark. The tryptic peptides were separated by reverse phase HPLC (Thermo Ultimate 3000) using a Thermo PepMap RSLC C18 column (2-um tip, 75umx50cm PN# ES903) over a 90 minute gradient before nano electrospray using a Orbitrap Exploris 480 mass spectrometer (Thermo). Solvent A was 0.1% formic acid in water and solvent B was 0.1% formic acid in acetonitrile. The gradient conditions were 1% B (0-10 min at 300nL/min) 1% B (10-15 min, 300 nL/min to 200 nL/min) 1-7% B (15-20 min, 200nL/min), 7-25% B (20-54.8 min, 200nL/min), 25-36 B (54.8-65 min, 200nL/min), 36-80% B (65-65.5 min, 200 nL/min), 80% B (65.5-70 min, 200nL/min), 80-1% B (70-70.1 min, 200nL/min), 1% B (70.1-90 min, 200nL/min). The Thermo Orbitrap Exploris 480 mass spectrometer was operated in a data-dependent mode. The parameters for the full scan MS were: resolution of 120,000 across 375-1600 m/z and maximum IT 25 ms. The full MS scan was followed by MS/MS for as many precursor ions in a two second cycle with a NCE of 28, dynamic exclusion of 20 s and resolution of 30,000. Raw mass spectral data files (.raw) were searched using Sequest HT in Proteome Discoverer (Thermo) against a *Saccharomyces cerevisiae* database (Uniprot) and a contaminates database (made in house) with the following search parameters: 10 ppm mass tolerance for precursor ions; 0.02 Da for fragment ion mass tolerance; 2 missed cleavages of trypsin; fixed modification were carbamidomethylation of cysteine, variable modifications were methionine oxidation, methionine loss at the N-terminus of the protein, acetylation of the N-terminus of the protein and also Met-loss plus acetylation of the protein N-terminus.

#### Unbiased matching of density map to models

The unbiased matching of density maps to protein models was performed using a modified method as described previously ^47^. Briefly, to identify the density associated with the FRI domain in the PpFz1-ωRNA-target DNA ternary complex, the target density was extracted using UCSF ChimeraX 1.7. The PDB library of the yeast proteome, containing 6,039 proteins, was downloaded from the AlphaFold2 database ^42^. The unbiased matching was conducted using the COLORES program (part of the Situs package; see Data and Code Availability section in the STAR Methods) ^53,55^. The matching was scored and ranked based on cross-correlation scores, and the top 50 hits were individually inspected alongside the target densities in UCSF ChimeraX 1.7.

#### Pull-down experiments of cyclophilins

To test the binding between the Fanzor RuvC Insertion (FRI) domain of PpFz1 and cyclophilins, eleven cyclophilin genes, including four homologs from *Saccharomyces cerevisiae* (ScCyp1, ScCyp2, ScCyp3, and ScCyp5), four homologs from *Parasitella parasitica* (PpCyp1, PpCyp2, PpCyp3, and PpCyp4), and three homologs from *humans* (hCypA, hCypF, and hCypH), were cloned with a N-terminal hexahistidine (His)-tag into multiple cloning site 1 (MCS1) of pETDuet-1 plasmid and a truncated version of the FRI domain was cloned into MCS2 with a N-terminal MBP tag. The expression plasmids of His-tagged cyclophilins and MBP-tagged FRI domain were transformed into E. coli Rosetta 2 competent cells (Novagen) and cultured at 37°C in Terrific Broth supplemented with 100 μg/mL ampicillin and 34 μg/mL chloramphenicol. When the OD600 reached 1.8, the protein expression was induced by the addition of 1 mM IPTG (Gold Biotechnology) and the *E. coli* cells were then allowed to grow further at 21°C overnight. The cells were harvested and resuspended in buffer A (50 mM Tris-HCl, pH 8.0, 150 mM NaCl, 15% [v/v] glycerol) supplemented with EDTA-free cOmplete protease inhibitor (Roche). The cells were lysed using a LM20 microfluidizer device (Microfluidics), and the cleared lysate was bound to Amylose Resin (New England Biolabs). The resin was washed with buffer A then eluted with buffer B (50 mM Tris-HCl, pH 8.0, 150 mM NaCl, 10 mM imidazole, 15% [v/v] glycerol) containing 40 mM maltose. The proteins were next bound to HisPur Ni-NTA magnetic beads (Thermo Fisher Scientific). The resin was washed with buffer B then eluted with buffer B supplemented with 500 mM imidazole. The resulting elution was analyzed by NuPAGE (Invitrogen) and eStain L1 Protein Staining System (GenScript).

#### Structural analysis of the lid region

A structural analysis was conducted focusing on the lid region of TnpB, Cas12, and Fanzor. Sequences for 59 TnpB representatives were extracted from the datasets of Nety et al. ^48^ and ISDra2 protein ^50^ were analyzed. Additionally, 23 representative Fanzors were sourced from Saito et al. ^51^, and 17 sequences of Cas12, one from each subtype, were selected (Data S1). Models for each protein were predicted using the ColabFold framework ^42,44^ with 40 recycles and 5 replicates. Models exhibiting the highest average predicted Local Distance Difference Test (pLDDT) scores were selected.

For analysis of the RuvC domain, the models were aligned to the RuvC domain extracted from ISDra2 (spanning residues P184 to I378; P184 is considered as position 1 for subsequent coordinates) using DeepAlign software (v.1.35) ^53^. Each structural alignment was manually inspected and adjusted as necessary within the PyMOL v2.5.4 using the Editing mode to insure the RuvC area is well superimposed. For each RuvC domain, pLDDT values were normalized to a scale of 0 to 100, where the minimum pLDDT value was set to 0 and the maximum pLDDT value was set to 100, ensuring consistent comparative analysis across all RuvC domains. Next, the focus was on the regions of the ISDra2 RuvC domain adjacent to the lid region (residues 96-117), which encompassed two secondary structure segments upstream (residues 74-89 and 90-95) and two downstream (residues 118-133 and 134-139). These segments were mapped across all proteins using the superimposition data, and normalized pLDDT values were extracted for each residue within these segments. The mean pLDDT for each segment was calculated and compared both across different proteins to assess variations between protein families and among segments within each protein to facilitate intra-family comparisons.

#### Quantification and Statistical Analysis

The statistical approach for each experiment is described in the figure legend or the STAR Methods section.

## Supplementary Material

Supplemental figures **Figure S1. Cryo-EM data processing for GtFz1, SpuFz1, and PpFz1. Related to** Figure 1.
(A) Flow chart of cryo-EM data analysis of GtFz1 sample 1 (native guide). Two ternary complexes (State II and III) and a binary complex were obtained.
(B) Flow chart of cryo-EM data analysis of GtFz1 sample 2 (PSP1 guide). The unsharpened map showing a DNA non-target strand (NTS) is stabilized by the protein.
(C) Representative cryo-EM image of GtFz1 from 12,579 movies.
(D) Representative and 2D averages of GtFz1 binary complex.
(E) Representative and 2D averages of GtFz1 ternary complex (State III).
(F) The ‘gold-standard’ FSC curves of the GtFz1 binary complex.
(G) The ‘gold-standard’ FSC curves of the GtFz1 ternary complex (State III).
(H) Flow chart of cryo-EM data analysis of SpuFz1. ds2: DNA target strand (TS) is partially modified and NTS is not modified. ds3: DNA TS is not modified and NTS is partially modified. ds5: DNA TS is fully modified and NTS is not modified. ds6: DNA TS is not modified and NTS is fully modified. The sequences of DNA substrate is shown in Figure S6.
(I) Representative cryo-EM image of SpuFz1-ds2 from 5,628 movies.
(J) Representative and 2D averages of SpuFz1.
(K) Flow chart of cryo-EM data analysis of PpFz1.
(L) Representative cryo-EM image of PpFz1 from 7,678 movies.
(M) Representative and 2D averages of PpFz1.
**Figure S2. The structure of PpFz1 in complex with yeast cyclophilin. Related to** Figure 1.
(A) Flow chart to identify the unknown density in the PpFz1 structure.
(B) Model of PpFz1 in complex with ScCyp1.
(C) Interface between PpFz and ScCyp1.
(D) Interaction between PpFz and ScCyp1.
(E) Pull-down experiment for FRI domain with 11 cyclophilin homologs.
(F) Sequence alignment of the 11 cyclophilins, including four homologs from *Saccharomyces cerevisiae* (ScCyp1, ScCyp2, ScCyp3, and ScCyp5), four homologs from *Parasitella parasitica* (PpCyp1, PpCyp2, PpCyp3, and PpCyp4), and three homologs from humans (hCypA, hCypF, and hCypH)
**Figure S3. Structural comparisons of the interactions between Fz1 and ωRNA. Related to** Figure 2.
(A) Electrostatic potential surface of Fz1s and the ωRNA binding surface.
(B) Structural comparison of the core regions of Fz-ωRNA complexes.
(C) Snapshots of the connections between ωRNA scaffolds and guide regions.
(D) Structures of the 5’ end of ωRNAs.
The REC domain is colored gray, the WED domain is colored yellow, the RuvC domain is colored cyan, the TNB domain is colored pink, ωRNA is colored purple, the DNA TS is colored red, and the DNA NTS is colored blue.
**Figure S4. DNA recognition of Fz1. Related to** Figure 3.
(A) Top view of Fz1 ternary complexes illustrating the bending of DNA at various angles. Fz1 proteins are colored white; DNA is colored red; and ωRNA is colored purple.
(B) TAM recognition by the REC and WED domains. The non-conserved structure of the helix portion is highlighted in yellow; the TAM sequence is colored pink; the REC domain is colored gray. A loop in GtFz1 and PpFz1 pushes the TAM duplex, causing the DNA angle to differ from that in SpuFz1.
(C) TAM sequence of PpFz1.
(D) In vitro cleavage assay of GtFz1 mutants to validate the interactions of TAM recognition.
(E) In vitro cleavage assay of GtFz1-mediated dsDNA cleavage for TAM variant. Ctrl indicates the DNA substrate with WT TAM.
(F) In vitro cleavage assay of GtFz1 mutants to validate the interactions between ωRNA and DNA.
(G) In vitro cleavage assay of GtFz1-mediated dsDNA cleavage for DNA variants to test the mismatch tolerance of the guide/DNA duplex.
(H) Interactions at the 3rd base pair (bp) of the GtFz1 heteroduplex.
(I) EM density and model of SpuFz1 state IV ωRNA and target DNA. The structure of SpuFz1 state IV reveals a 20-bp heteroduplex, with a 5-bp extension exposed to solvent.
**Figure S5. In vitro activity of GtFz1. Related to** Figure 4.
(A) Temperature dependence of GtFz1-mediated target dsDNA cleavage activity.
(B) GtFz1-mediated target dsDNA cleavage dependence on divalent metal ions. Target dsDNA substrates were column-purified after proteinase treatment and run on a 2% agarose gel. All experiments except those corresponding to this panel were performed with Mg2+
(C) Conserved catalytic motifs in the RuvC domain are shared among TnpB, Fz2, Fz1, and Cas12a.
(D) In vitro cleavage activity of AsCas12a, LbCas12a, and IsDra2TnpB harboring mutations in the catalytic motifs.
Figure S6. SpuFz exhibits dynamic cleavage sites for both TS and NTS. Related to Figure 6.
(A) Substrates (ds0 - 3) used for in vitro cleavage assays. Pink, TAM sequence; purple, guide sequence. The black box indicates the sequence has been phosphorothioate modified. The blue dashed line indicates the cleavage site on the NTS. The red dashed line indicates the cleavage site on the TS.
(B) SpuFz1-mediated target dsDNA cleavage with substrates from (A). Red arrows indicate the cleaved TS products, blue arrows indicate the cleaved NTS products. Cleavage was visualized with a 15% TB-Urea gel and stained by SYBR Gold.
(C) Structure of SpuFz1 State III (substrate ds2, TS partially modified and NTS is not modified). Overall structure showing dsDNA is bound to the large cleft formed by the REC/RuvC/TNB domains (left). Electrostatic potential surface of SpuFz1 showing the large cleft accommodating dsDNA binding (middle). EM density and model of dsDNA is shown (right).
(D) Structure of SpuFz1 State IV (substrate ds3, TS not modified and NTS is partially modified). Overall structure showing dsDNA is unwound and bound to the large cleft formed by the REC/RuvC/TNB domains (left). Electrostatic potential surface of SpuFz1 showing the large cleft accommodating dsDNA binding (middle). EM density and model of dsDNA is shown (right).
(E) Substrates (ds4 - 6) used for in vitro cleavage assays. Pink, TAM sequence; purple, guide sequence. The black box indicates the sequence has been phosphorothioate modified. The blue dashed line indicates the cleavage site on NTS. The red dashed line indicates the cleavage site on TS.
(F) SpuFz1-mediated target dsDNA cleavage with substrates from (E). Red arrows indicate the cleaved TS products, blue arrows indicate the cleaved NTS products. Cleavage was visualized with a 15% TB-Urea gel and stained by SYBR Gold. An excess of substrate was added and the sample was taken from the molecular sieve so that the uncleaved bands are significantly more obvious than the cleaved bands.
**Figure S7. Comparison of the lid structures in Fzs, TnpB, and Cas12s. Related to** Figure 7.
The lid structure is shown as a surface model and colored in blue. The dashed line indicates the guide/DNA duplex. The red dashed circle indicates the catalytic sites.
**Figure S8. Comparison of the R-loop structures of GtFz1, SpuFz1, and GtFz1. Related to** Figure 5–7.
(A) EM map of GtFz1 ternary complex showing the NTS binding to the RuvC active core. Gray, GtFz1; red, DNA; purple, ωRNA.
(B) Electrostatic potential mapping in GtFz1 illustrates the DNA binding channel.
(C) EM map of the SpuFz1 ternary complex showing the NTS binding to the RuvC active core.
(D) Electrostatic potential mapping of SpuFz1 illustrates the DNA binding channel.
(E) EM map of the PpFz1 ternary complex showing the NTS binding to the RuvC active core.
(F) Electrostatic potential mapping of PpFz1 illustrates the DNA binding channel.

Table S3 **Table S3. ωRNA, protein, and TAM sequence information for GtFz1, SpuFz1, and PpFz1. Related to** Figure 1 and 2.

Table S2 **Table S2. Mass spectrometry data from the PpFz1-ωRNA-target DNA complex sample. Related to** Figure 1 and Figure S2.
Protein candidates ranked by total spectrum count. *Saccharomyces cerevisiae* Cyclophilin1 (ScCyp1) was identified and used for model building of the cyclophilin-bound PpFz1-ωRNA-target DNA structure.

Data S1 **Data S1. List and the predicted Local Distance Difference Test (pLDDT) values of TnpBs, Cas12s, and Fzs used for lid analysis. Related to** Figure 7.

Table S1 **Table S1. Cryo-EM data collection, refinement validation statistics. Related to** Figure 1.

Movie S1 **Movie S1. 3D Variability Analysis (3DVA) reveals the conformational dynamics of SL1 and the RuvC/TNB domains of GtFz1. Related to** Figure 5.

Movie S2 **Movie S2. 3D Variability Analysis (3DVA) reveals the stable structure of SL1 and the RuvC/TNB domains of SpuFz1. Related to** Figure 5.

Supplemental materials

## Figures and Tables

**Table:** Key resources table

REAGENT or RESOURCE	SOURCE	IDENTIFIER
Antibodies		
		
		
		
		
		
Bacterial and virus strains		
One Shot Stbl3 Chemically Competent E. coli	ThermoFischer	C737303
Rosetta^™^ 2(DE3)pLysS Competent Cells E. coli	Novagen	71403
		
		
		
Biological samples		
		
		
		
		
		
Chemicals, peptides, and recombinant proteins		
Q5 High-Fidelity 2X Master Mix	New England Biolabs	M0492
KAPA HiFi HotStart ReadyMix	Roche	KK2602
NEBNext High-Fidelity 2X PCR Master Mix	New England Biolabs	M0541
KLD Enzyme Mix	New England Biolabs	M0554S
2x Phanta Max Master Mix	Vazyme	P515-02
E-Gel EX Agarose Gels, 1%	ThermoFischer	G401001
E-Gel EX Agarose Gels, 2%	ThermoFischer	G401002
E-Gel EX Agarose Gels, 4%	ThermoFischer	G401004
Novex^™^ TBE-Urea Gels, 15%	ThermoFischer	EC68852BOX
Novex^™^ TBE Gels, 6%	ThermoFischer	EC62655BOX
Invitrogen novex TBE Running Buffer (5X)	ThermoFischer	LC6675
QIAquick PCR Purification Kit	QIAGEN	28106
QIAprep Spin Miniprep Kit	QIAGEN	27106
Gibson Assembly^®^ Master Mix	New England Biolabs	E2611
NEBuilder^®^ HiFi DNA Assembly Master Mix	New England Biolabs	E2621
Isopropyl-β-D-thiogalactopyranoside	Goldbio	I2481C
TRI Reagent	ZYMO RESEARCH	R2050-1-200
RNA Clean & Concentrator-25	ZYMO RESEARCH	R1017
DNase I (RNase-free)	New England Biolabs	M0303
RNase A	QIAGEN	19101
SUPERasedIn RNase Inhibitor (20 U/mL)	ThermoFischer	AM2694
AMPure XP for PCR Purification	Beckman Coulter	A63881
Tn5	Schmid-Burgk et al.^28^	N/A
Protease Inhibitor Cocktail (EDTA-Free, 100X in DMSO)	MedChem Express	HY-K0010
EDTA-free cOmplete protease inhibitor	Roche	11836153001
Amicon Ultra-15 Centrifugal Filter Units 50kDa NMWL	Millipore sigma	UFC905024
NuPAGE 4-12% Bis-Tris Protein Gels, 1.0 mm, 12-well	ThermoFischer	NP0322BOX
NuPAGE LDS Sample Buffer (4X)	ThermoFischer	NP0007
Ampicillin, sodium salt	AmericanBio	Ab00115
Kanamycin sulfate from Streptomyces kanamyceticus	Sigma	K4000
HEPES	Sigma-Aldrich	PHG0001
Sodium chloride	Sigma-Aldrich	71376
Magnesium chloride hexahydrate	Sigma-Aldrich	M0250
TCEP hydrochloride	Hampton Research	HR2-801
TALON resin	Takara Clontech	635504
Amylose Resin	New England Biolabs	E8021L
D-(+)-Maltose monohydrate	Sigma-Aldrich	M9171
Critical commercial assays		
Qubit 1X dsDNA HS (HighSensitivity) Assay Kit	ThermoFischer	Q33231
eStain L1 Protein Staining System	GenScript	N/A
MiSeq Reagent Kits v2	Illumina	MS-102
		
		
Deposited data		
GtFanzor-omegaRNA state 1 EM map	This work	EMDB: EMD-45516
GtFanzor-omegaRNA-targetDNA state 2 EM map	This work	EMDB: EMD-45517
GtFanzor-omegaRNA-targetDNA state 3 EM map	This work	EMDB: EMD-45518
SpuFanzor-omegaRNA-targetDNA state 1 EM map	This work	EMDB: EMD-45519
SpuFanzor-omegaRNA-targetDNA state 2 EM map	This work	EMDB: EMD-45520
SpuFanzor-omegaRNA-targetDNA state 3 EM map	This work	EMDB: EMD-45521
SpuFanzor-omegaRNA-targetDNA state 4 EM map	This work	EMDB: EMD-45522
SpuFanzor-omegaRNA-targetDNA state 5 EM map	This work	EMDB: EMD-45523
SpuFanzor-omegaRNA-targetDNA state 6 EM map	This work	EMDB: EMD-45524
PpFanzor-omegaRNA-targetDNA state 1 EM map	This work	EMDB: EMD-45525
PpFanzor-omegaRNA-targetDNA state 2 EM map	This work	EMDB: EMD-45526
PpFanzor-omegaRNA-targetDNA state 3 EM map	This work	EMDB: EMD-45527
PpFanzor-omegaRNA-targetDNA state 4 EM map	This work	EMDB: EMD-45528
GtFanzor-omegaRNA state 1 model	This work	PDB: 9CER
GtFanzor-omegaRNA-targetDNA state 2 model	This work	PDB: 9CES
GtFanzor-omegaRNA-targetDNA state 3 model	This work	PDB: 9CET
SpuFanzor-omegaRNA-targetDNA state 1 model	This work	PDB: 9CEU
SpuFanzor-omegaRNA-targetDNA state 2 model	This work	PDB: 9CEV
SpuFanzor-omegaRNA-targetDNA state 3 model	This work	PDB: 9CEW
SpuFanzor-omegaRNA-targetDNA state 4 model	This work	PDB: 9CEX
SpuFanzor-omegaRNA-targetDNA state 5 model	This work	PDB: 9CEY
SpuFanzor-omegaRNA-targetDNA state 6 model	This work	PDB: 9CEZ
PpFanzor-omegaRNA-targetDNA state 1 model	This work	PDB: 9CF1
PpFanzor-omegaRNA-targetDNA state 2 model	This work	PDB: 9CF2
PpFanzor-omegaRNA-targetDNA state 3 model	This work	PDB: 9CF3
PpFanzor-omegaRNA-targetDNA state 4 model	This work	PDB:
Experimental models: Cell lines		
Saccharomyces cerevisiae Meyen ex E.C. Hansen BCY123	Galej et al. ^29^	N/A
		
		
		
		
Experimental models: Organisms/strains		
		
		
		
		
		
		
Oligonucleotides		
GAGAACATTGACAGAGTAGAGTTTAATGCTCTTAAATGCCCGGGTACC	Azenta Life Science	GtFz_ntv_TS
GGTACCCGGGCATTTAAG	Azenta Life Science	GtFz_ntv_NTS
GACCATGATTACGCCAAGCTTT*T*T*A*A*C*A*G*T*G*G*C*C*T*T*A*T*T*A*A*ATGACTTCTCCTTAAATGCCCGGGTACCGAGCTCGAATTC	IDT	GtFz_PSP1_TS
GAATTCGAGCTCGGTACCCGGGCATTTAAGGAGAAGTCATT*T*A*A*T*A*A*G*G*C*C*A*C*T*G*T*T*A*A*A*AAGCTTGGCGTAATCATGGTC	IDT	GtFz_PSP1_NTS
ATTCGAGCTCGGTACCCGGGCATATCTATAGGTTATGAAATCAAATTACAAATA	IDT	SpuFz_TS
TATTTGTAATTTGATTTCATAACCTATAGATATGCCCGGGTACCGAGCTCGAAT	IDT	SpuFz_NTS
ATTCGAGCTCGGTACCCGGGCATATCTATAGGTTATG*A*A*A*T*C*A*A*A*T*TACAAATA	IDT	SpuFz_TSpm
TATTTG*T*A*A*T*T*T*G*A*T*T*TCATAACCTATAGATATGCCCGGGTACCGAGCTCGAAT	IDT	SpuFz_NTSpm
AT*T*C*G*A*G*C*T*C*G*G*T*A*C*C*C*G*G*G*C*A*T*A*T*C*T*A*T*A*G*G*T*T*A*T*G*A*A*A*T*C*A*A*A*T*T*A*C*A*A*A*T*A	IDT	SpuFz_TSfm
TA*T*T*T*G*T*A*A*T*T*T*G*A*T*T*T*C*A*T*A*A*C*C*T*A*T*A*G*A*T*A*T*G*C*C*C*G*G*G*T*A*C*C*G*A*G*C*T*C*G*A*A*T	IDT	SpuFz_NTSfm
TACCCGGGATAAACATCCAGCAAACAGAGCTCGTTCAAAAACTAATTTCCTTTTGAC	Azenta Life Science	PpFz_TS
GTCAAAAGGAAATTAGTTTTTGAACGAGCTCTGTTTGCTGGATGTTTATCCCGGGTA	Azenta Life Science	PpFz_NTS
Recombinant DNA		
		
		
		
		
		
Software and algorithms		
Geneious Prime	Dotmatics	https://www.geneious.com/
Image Lab 1.6	Bio-Rad	https://www.bio-rad.com/
PyMOL 2.5	Schrödinger	https://pymol.org/2/
Weblogos	Grooks et al.^31^	https://weblogo.berkeley.edu/
Prism 8	GraphPad	https://www.graphpad.com/scientific-software/prism/
Leginon 3.6	Suloway et al.^30^	https://emg.nysbc.org/redmine/projects/leginon/wiki/Leginon_Homepage
MotionCor2	Zheng et al.^34^	https://emcore.ucsf.edu/ucsf-software
CTFFIND4	Rohou et al.^37^	https://grigoriefflab.umassmed.edu/ctffind4
Phenix 1.2.1	Liebschner et al.^7^	https://phenix-online.org/
UCSF ChimeraX 1.7	Goddard et al.^8^	https://www.cgl.ucsf.edu/chimerax/
UCSF Chimera 1.16	Pettersen et al.^9^	https://www.cgl.ucsf.edu/chimera/
Coot 0.9.8.1	Emsley et al. ^43^	https://www2.mrc-lmb.cam.ac.uk/personal/pemsley/coot/
ISOLDE 1.7	Tristan et al.^41^	https://tristanic.github.io/isolde/
CryoSPARC 4.3	Punjani et al.^32^	https://cryosparc.com/
DeepEMhancer	Sanchez-Garcia et al.^38^	https://github.com/rsanchezgarc/deepEMhancer
Relion 4.0	Kimanius et al.^14^	https://relion.readthedocs.io/
MolProbity	Chen et al.^46^	http://molprobity.biochem.duke.edu/
AlphaFold2	Jumper et al.^42^	https://alphafold.ebi.ac.uk/
Situs	Wriggers et al.^53^	https://situs.biomachina.org/fguide.html
RNAcomposer	Antczak et al.^39^	https://rnacomposer.cs.put.poznan.pl/
Adobe Illustrator CC	Adobe	https://www.adobe.com
Other		
Superdex 200 Increase 10/300 GL	Cytiva	28990944
Superose 6 Increase 5/150 GL	Cytiva	29091597
Superose 6 Increase 10/300 column	Cytiva	29091596
CryoMatrix R1.2/1.3 300-mesh gold holey grids with amorphous alloy film	Zhenjiang Lehua Technology Co., Ltd	M024-Au300-R12/13
UltrAuFoil R 1.2/1.3, 300 mesh, Gold	Quantifoil^®^	Q350AR13A
Quantifoil^®^ Holey Carbon Film R 1.2/1.3 Gold 300 Grid	Quantifoil^®^	50-209-1364
		
		
		

## References

[1] Jiang K, Lim J, Sgrizzi S, Trinh M, Kayabolen A, Yutin N, Bao W, Kato K, Koonin EV, Gootenberg JS, (2023). Programmable RNA-guided DNA endonucleases are widespread in eukaryotes and their viruses. Sci Adv 9, eadk0171. 37756409 10.1126/sciadv.adk0171 PMC10530073

[2] Saito M, Xu P, Faure G, Maguire S, Kannan S, Altae-Tran H, Vo S, Desimone A, Macrae RK, and Zhang F (2023). Fanzor is a eukaryotic programmable RNA-guided endonuclease. Nature 620, 660–668. 37380027 10.1038/s41586-023-06356-2 PMC10432273

[3] Bao W, and Jurka J (2013). Homologues of bacterial TnpB_IS605 are widespread in diverse eukaryotic transposable elements. Mob. DNA 4, 12. 23548000 10.1186/1759-8753-4-12 PMC3627910

[4] Altae-Tran H, Kannan S, Demircioglu FE, Oshiro R, Nety SP, McKay LJ, Dlakić M, Inskeep WP, Makarova KS, Macrae RK, (10/2021). The widespread IS200/IS605 transposon family encodes diverse programmable RNA-guided endonucleases. Science 374, 57–65. 34591643 10.1126/science.abj6856 PMC8929163

[5] Yoon PH, Skopintsev P, Shi H, Chen L, Adler BA, Al-Shimary M, Craig RJ, Loi KJ, DeTurk EC, Li Z, (2023). Eukaryotic RNA-guided endonucleases evolved from a unique clade of bacterial enzymes. Nucleic Acids Res. 51, 12414–12427. 37971304 10.1093/nar/gkad1053 PMC10711439

[6] Altae-Tran H, Shmakov SA, Makarova KS, Wolf YI, Kannan S, Zhang F, and Koonin EV (2023). Diversity, evolution, and classification of the RNA-guided nucleases TnpB and Cas12. Proc. Natl. Acad. Sci. U. S. A 120, e2308224120. 37983496 10.1073/pnas.2308224120 PMC10691335

[7] Shmakov S, Abudayyeh OO, Makarova KS, Wolf YI, Gootenberg JS, Semenova E, Minakhin L, Joung J, Konermann S, Severinov K, (2015). Discovery and Functional Characterization of Diverse Class 2 CRISPR-Cas Systems. Mol. Cell 60, 385–397. 26593719 10.1016/j.molcel.2015.10.008 PMC4660269

[8] Nakagawa R, Hirano H, Omura SN, Nety S, Kannan S, Altae-Tran H, Yao X, Sakaguchi Y, Ohira T, Wu WY, (2023). Cryo-EM structure of the transposon-associated TnpB enzyme. Nature 616, 390–397. 37020030 10.1038/s41586-023-05933-9 PMC10097598

[9] Sasnauskas G, Tamulaitiene G, Druteika G, Carabias A, Silanskas A, Kazlauskas D, Venclovas Č, Montoya G, Karvelis T, and Siksnys V (2023). TnpB structure reveals minimal functional core of Cas12 nuclease family. Nature 616, 384–389. 37020015 10.1038/s41586-023-05826-x

[10] Wang P, and Heitman J (2005). The cyclophilins. Genome Biol. 6, 1–6. 10.1186/gb-2005-6-7-226 PMC1175980 15998457

[11] Yamano T, Nishimasu H, Zetsche B, Hirano H, Slaymaker IM, Li Y, Fedorova I, Nakane T, Makarova KS, Koonin EV, (2016). Crystal Structure of Cpf1 in Complex with Guide RNA and Target DNA. Cell 165, 949–962. 27114038 10.1016/j.cell.2016.04.003 PMC4899970

[12] Yang H, Gao P, Rajashankar KR, and Patel DJ (2016). PAM-Dependent Target DNA Recognition and Cleavage by C2c1 CRISPR-Cas Endonuclease. Cell 167, 1814–1828.e12. 27984729 10.1016/j.cell.2016.11.053 PMC5278635

[13] Kurihara N, Nakagawa R, Hirano H, Okazaki S, Tomita A, Kobayashi K, Kusakizako T, Nishizawa T, Yamashita K, Scott DA, (2022). Structure of the type V-C CRISPR-Cas effector enzyme. Mol. Cell 82, 1865–1877.e4. 35366394 10.1016/j.molcel.2022.03.006 PMC9522604

[14] Liu J-J, Orlova N, Oakes BL, Ma E, Spinner HB, Baney KLM, Chuck J, Tan D, Knott GJ, Harrington LB, (2019). Author Correction: CasX enzymes comprise a distinct family of RNA-guided genome editors. Nature 568, E8–E10. 30944483 10.1038/s41586-019-1084-8

[15] Zhang H, Li Z, Xiao R, and Chang L (2020). Mechanisms for target recognition and cleavage by the Cas12i RNA-guided endonuclease. Nat. Struct. Mol. Biol 27, 1069–1076. 32895556 10.1038/s41594-020-0499-0 PMC8256696

[16] Al-Shayeb B, Skopintsev P, Soczek KM, Stahl EC, Li Z, Groover E, Smock D, Eggers AR, Pausch P, Cress BF, (2022). Diverse virus-encoded CRISPR-Cas systems include streamlined genome editors. Cell 185, 4574–4586.e16. 36423580 10.1016/j.cell.2022.10.020

[17] Shi Y-J, Duan M, Ding J-M, Wang F-Q, Bi L-L, Zhang C-X, Zhang Y-Z, Duan J-Y, Huang A-H, Lei X-L, (2022). DNA topology regulates PAM-Cas9 interaction and DNA unwinding to enable near-PAMless cleavage by thermophilic Cas9. Mol. Cell 82, 4160–4175.e6. 36272409 10.1016/j.molcel.2022.09.032

[18] Swarts DC (2019). Making the cut(s): how Cas12a cleaves target and non-target DNA. Biochem. Soc. Trans 47, 1499–1510. 31671185 10.1042/BST20190564

[19] Swarts DC, and Jinek M (02/2019). Mechanistic Insights into the cis- and trans-Acting DNase Activities of Cas12a. Mol. Cell 73, 589–600.e4. 30639240 10.1016/j.molcel.2018.11.021 PMC6858279

[20] Jeon Y, Choi YH, Jang Y, Yu J, Goo J, Lee G, Jeong YK, Lee SH, Kim I-S, Kim J-S, (2018). Direct observation of DNA target searching and cleavage by CRISPR-Cas12a. Nat. Commun 9, 2777. 30018371 10.1038/s41467-018-05245-x PMC6050341

[21] Paul B, and Montoya G (2020-2). CRISPR-Cas12a: Functional overview and applications. Biomed. J 43, 8–17. 32200959 10.1016/j.bj.2019.10.005 PMC7090318

[22] Pacesa M, Loeff L, Querques I, Muckenfuss LM, Sawicka M, and Jinek M (2022). R-loop formation and conformational activation mechanisms of Cas9. Nature 609, 191–196. 36002571 10.1038/s41586-022-05114-0 PMC9433323

[23] Cofsky JC, Karandur D, Huang CJ, Witte IP, Kuriyan J, and Doudna JA (2020). CRISPR-Cas12a exploits R-loop asymmetry to form double-strand breaks. Elife 9, e55143. 32519675 10.7554/eLife.55143 PMC7286691

[24] Schultze K, Schimek C, Wöstemeyer J, and Burmester A (2005). Sexuality and parasitism share common regulatory pathways in the fungus Parasitella parasitica. Gene 348, 33–44. 15777660 10.1016/j.gene.2005.01.007

[25] Nigro P, Pompilio G, and Capogrossi MC (2013). Cyclophilin A: a key player for human disease. Cell Death Dis. 4, e888. 24176846 10.1038/cddis.2013.410 PMC3920964

[26] Rajiv C, and Davis TL (2018). Structural and Functional Insights into Human Nuclear Cyclophilins. Biomolecules 8. 10.3390/biom8040161. PMC6315705 30518120

[27] Stella S, Alcón P, and Montoya G (2017). Structure of the Cpf1 endonuclease R-loop complex after target DNA cleavage. Nature 546, 559–563. 28562584 10.1038/nature22398

[28] Schmid-Burgk JL, Gao L, Li D, Gardner Z, Strecker J, Lash B, and Zhang F (2020). Highly Parallel Profiling of Cas9 Variant Specificity. Mol. Cell 78, 794–800.e8. 32187529 10.1016/j.molcel.2020.02.023 PMC7370240

[29] Galej WP, Oubridge C, Newman AJ, and Nagai K (2013). Crystal structure of Prp8 reveals active site cavity of the spliceosome. Nature 493, 638–643. 23354046 10.1038/nature11843 PMC3672837

[30] Suloway C, Pulokas J, Fellmann D, Cheng A, Guerra F, Quispe J, Stagg S, Potter CS, and Carragher B (2005). Automated molecular microscopy: the new Leginon system. J. Struct. Biol 151, 41–60. 15890530 10.1016/j.jsb.2005.03.010

[31] Crooks GE, Hon G, Chandonia J-M, and Brenner SE (2004). WebLogo: a sequence logo generator. Genome Res. 14, 1188–1190. 15173120 10.1101/gr.849004 PMC419797

[32] Punjani A, Rubinstein JL, Fleet DJ, and Brubaker MA (2017). cryoSPARC: algorithms for rapid unsupervised cryo-EM structure determination. Nat. Methods 14, 290–296. 28165473 10.1038/nmeth.4169

[33] Scheres SHW (2012). RELION: implementation of a Bayesian approach to cryo-EM structure determination. J. Struct. Biol 180, 519–530. 23000701 10.1016/j.jsb.2012.09.006 PMC3690530

[34] Zheng SQ, Palovcak E, Armache J-P, Verba KA, Cheng Y, and Agard DA (2017). MotionCor2: anisotropic correction of beam-induced motion for improved cryo-electron microscopy. Nat. Methods 14, 331–332. 28250466 10.1038/nmeth.4193 PMC5494038

[35] Tegunov D, and Cramer P (2019). Real-time cryo-electron microscopy data preprocessing with Warp. Nat. Methods 16, 1146–1152. 31591575 10.1038/s41592-019-0580-y PMC6858868

[36] Punjani A, Zhang H, and Fleet DJ (2020). Non-uniform refinement: adaptive regularization improves single-particle cryo-EM reconstruction. Nat. Methods 17, 1214–1221. 33257830 10.1038/s41592-020-00990-8

[37] Rohou A, and Grigorieff N (2015). CTFFIND4: Fast and accurate defocus estimation from electron micrographs. J. Struct. Biol 192, 216–221. 26278980 10.1016/j.jsb.2015.08.008 PMC6760662

[38] Sanchez-Garcia R, Gomez-Blanco J, Cuervo A, Carazo JM, Sorzano COS, and Vargas J (2021). DeepEMhancer: a deep learning solution for cryo-EM volume post-processing. Commun Biol 4, 874. 34267316 10.1038/s42003-021-02399-1 PMC8282847

[39] Antczak M, Popenda M, Zok T, Sarzynska J, Ratajczak T, Tomczyk K, Adamiak RW, and Szachniuk M (2016). New functionality of RNAComposer: an application to shape the axis of miR160 precursor structure. Acta Biochim. Pol 63, 737–744. 27741327 10.18388/abp.2016_1329

[40] Pettersen EF, Goddard TD, Huang CC, Meng EC, Couch GS, Croll TI, Morris JH, and Ferrin TE (2021). UCSF ChimeraX: Structure visualization for researchers, educators, and developers. Protein Sci. 30, 70–82. 32881101 10.1002/pro.3943 PMC7737788

[41] Croll TI (2018). ISOLDE: a physically realistic environment for model building into low-resolution electron-density maps. Acta Crystallogr D Struct Biol 74, 519–530. 29872003 10.1107/S2059798318002425 PMC6096486

[42] Jumper J, Evans R, Pritzel A, Green T, Figurnov M, Ronneberger O, Tunyasuvunakool K, Bates R, Žídek A, Potapenko A, (2021). Highly accurate protein structure prediction with AlphaFold. Nature 596, 583–589. 34265844 10.1038/s41586-021-03819-2 PMC8371605

[43] Emsley P, and Cowtan K (2004). Coot: model-building tools for molecular graphics. Acta Crystallogr. D Biol. Crystallogr 60, 2126–2132. 15572765 10.1107/S0907444904019158

[44] Mirdita M, Schütze K, Moriwaki Y, Heo L, Ovchinnikov S, and Steinegger M (2022). ColabFold: making protein folding accessible to all. Nat. Methods 19, 679–682. 35637307 10.1038/s41592-022-01488-1 PMC9184281

[45] Adams PD, Afonine PV, Bunkóczi G, Chen VB, Davis IW, Echols N, Headd JJ, Hung L-W, Kapral GJ, Grosse-Kunstleve RW, (2010). PHENIX: a comprehensive Python-based system for macromolecular structure solution. Acta Crystallogr. D Biol. Crystallogr 66, 213–221. 20124702 10.1107/S0907444909052925 PMC2815670

[46] Chen VB, Arendall WB 3rd, Headd JJ, Keedy DA, Immormino RM, Kapral GJ, Murray LW, Richardson JS, and Richardson DC (2010). MolProbity: all-atom structure validation for macromolecular crystallography. Acta Crystallogr. D Biol. Crystallogr 66, 12–21. 20057044 10.1107/S0907444909042073 PMC2803126

[47] Chen Z, Shiozaki M, Haas KM, Skinner WM, Zhao S, Guo C, Polacco BJ, Yu Z, Krogan NJ, Lishko PV, (2023). De novo protein identification in mammalian sperm using in situ cryoelectron tomography and AlphaFold2 docking. Cell 186, 5041–5053.e19. 37865089 10.1016/j.cell.2023.09.017 PMC10842264

[48] Nety SP, Altae-Tran H, Kannan S, Demircioglu FE, Faure G, Hirano S, Mears K, Zhang Y, Macrae RK, and Zhang F (2023). The Transposon-Encoded Protein TnpB Processes Its Own mRNA into ωRNA for Guided Nuclease Activity. CRISPR J 6, 232–242. 37272862 10.1089/crispr.2023.0015 PMC10278001

[49] Karvelis T, Druteika G, Bigelyte G, Budre K, Zedaveinyte R, Silanskas A, Kazlauskas D, Venclovas Č, and Siksnys V (2021). Transposon-associated TnpB is a programmable RNA-guided DNA endonuclease. Nature 599, 692–696. 34619744 10.1038/s41586-021-04058-1 PMC8612924

[50] Wang S, Ma J, Peng J, and Xu J (2013). Protein structure alignment beyond spatial proximity. Sci. Rep 3, 1448. 23486213 10.1038/srep01448 PMC3596798

[51] Liebschner D, Afonine PV, Baker ML, Bunkóczi G, Chen VB, Croll TI, Hintze B, Hung LW, Jain S, McCoy AJ, (2019). Macromolecular structure determination using X-rays, neutrons and electrons: recent developments in Phenix. Acta Crystallogr. D Struct. Biol 75, 861–877. 31588918 10.1107/S2059798319011471 PMC6778852

[52] Goddard TD, Huang CC, Meng EC, Pettersen EF, Couch GS, Morris JH, and Ferrin TE (2018). UCSF ChimeraX: Meeting modern challenges in visualization and analysis. Protein Sci. 27, 14–25. 28710774 10.1002/pro.3235 PMC5734306

[53] Wriggers W, Milligan RA, and McCammon JA (1999). Situs: A package for docking crystal structures into low-resolution maps from electron microscopy. J. Struct. Biol 125, 185–195. 10222274 10.1006/jsbi.1998.4080

[54] Pettersen EF, Goddard TD, Huang CC, Couch GS, Greenblatt DM, Meng EC, and Ferrin TE (2004). UCSF Chimera--a visualization system for exploratory research and analysis. J. Comput. Chem 25, 1605–1612. 15264254 10.1002/jcc.20084

[55] Wriggers W. (2012). Conventions and workflows for using Situs. Acta Crystallogr. D Biol. Crystallogr 68, 344–351. 22505255 10.1107/S0907444911049791 PMC3322594

[56] Kimanius D, Dong L, Sharov G, Nakane T, and Scheres SHW (2021). New tools for automated cryo-EM single-particle analysis in RELION-4.0. Biochem. J 478, 4169–4185. 34783343 10.1042/BCJ20210708 PMC8786306

