# Supplementary figures and images for: Structural Insights into the Diversity and DNA Cleavage Mechanism of Fanzor
Source: Cell. Author manuscript; Available in PMC 2024 Sep 25. (PMC11423790; doi:10.1016/j.cell.2024.07.050)

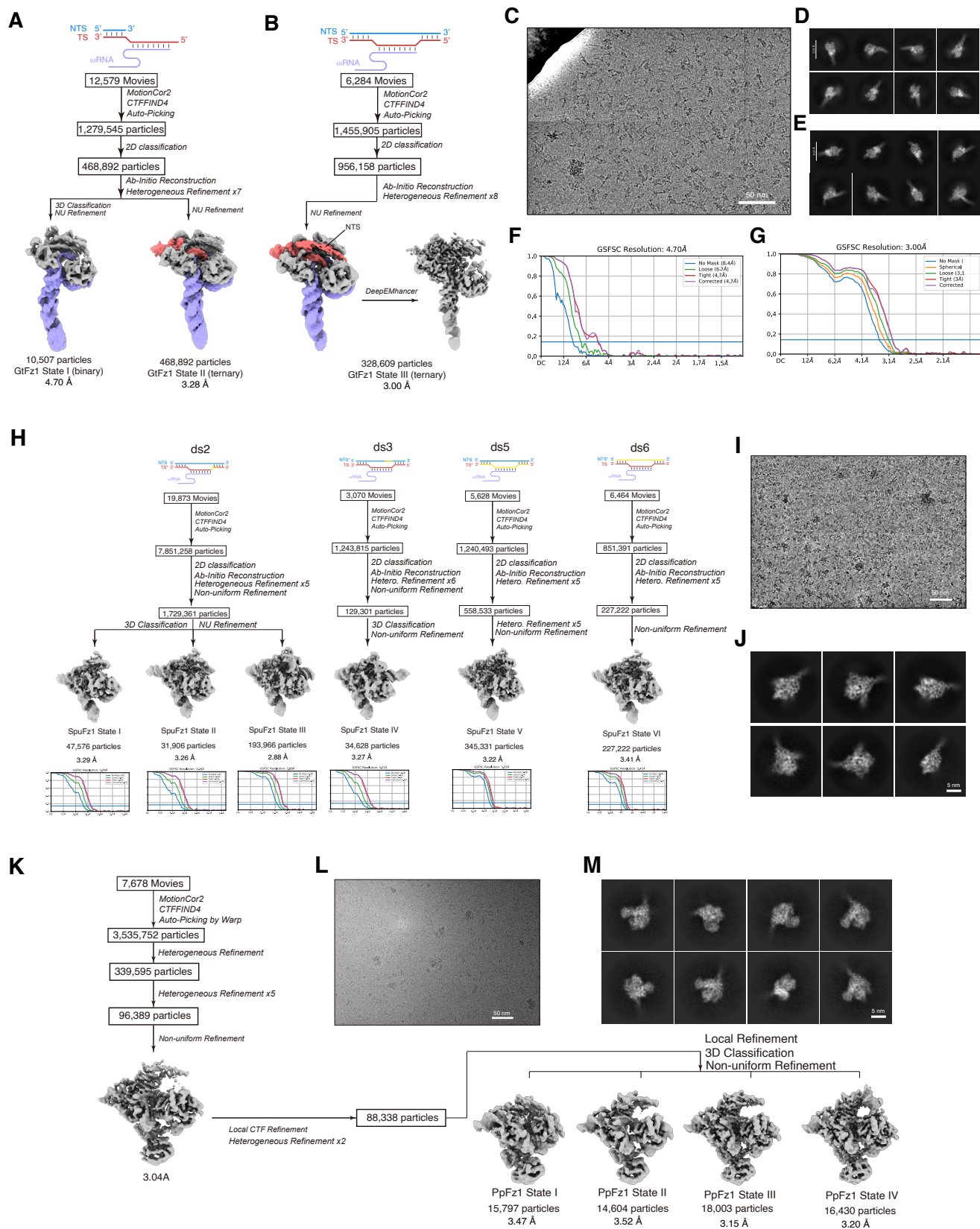

**Figure S1**

**A**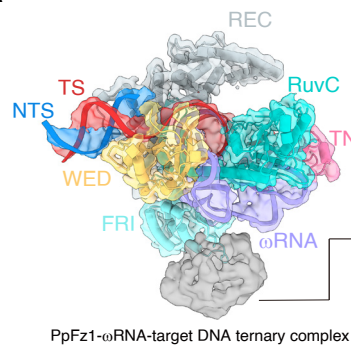**B**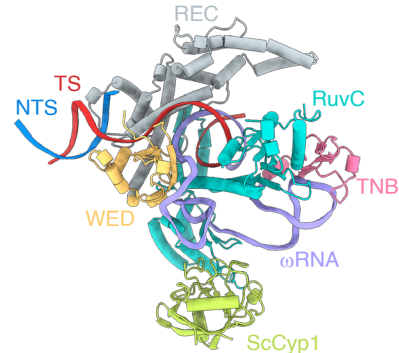**C**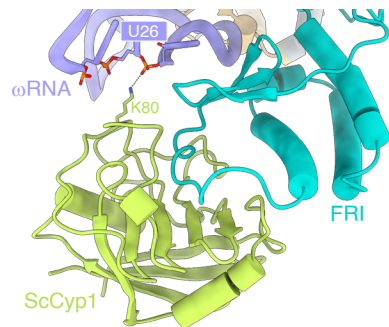**D**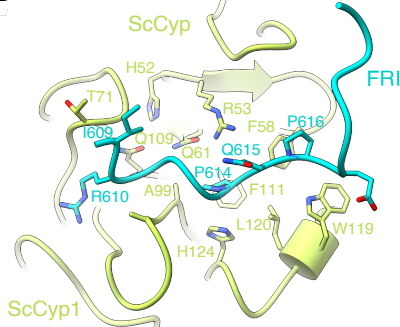**E**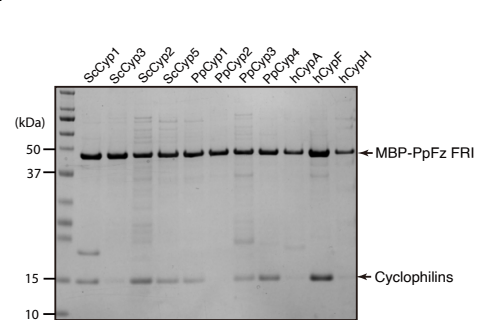**F**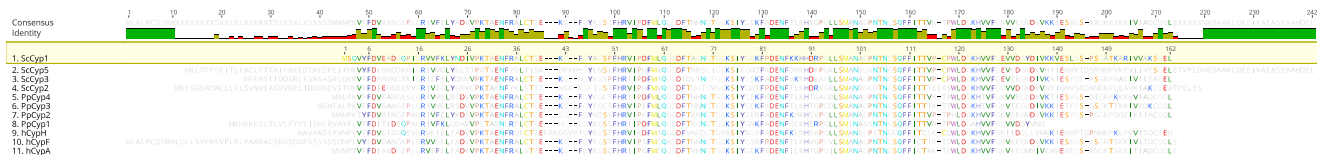**Figure S2**

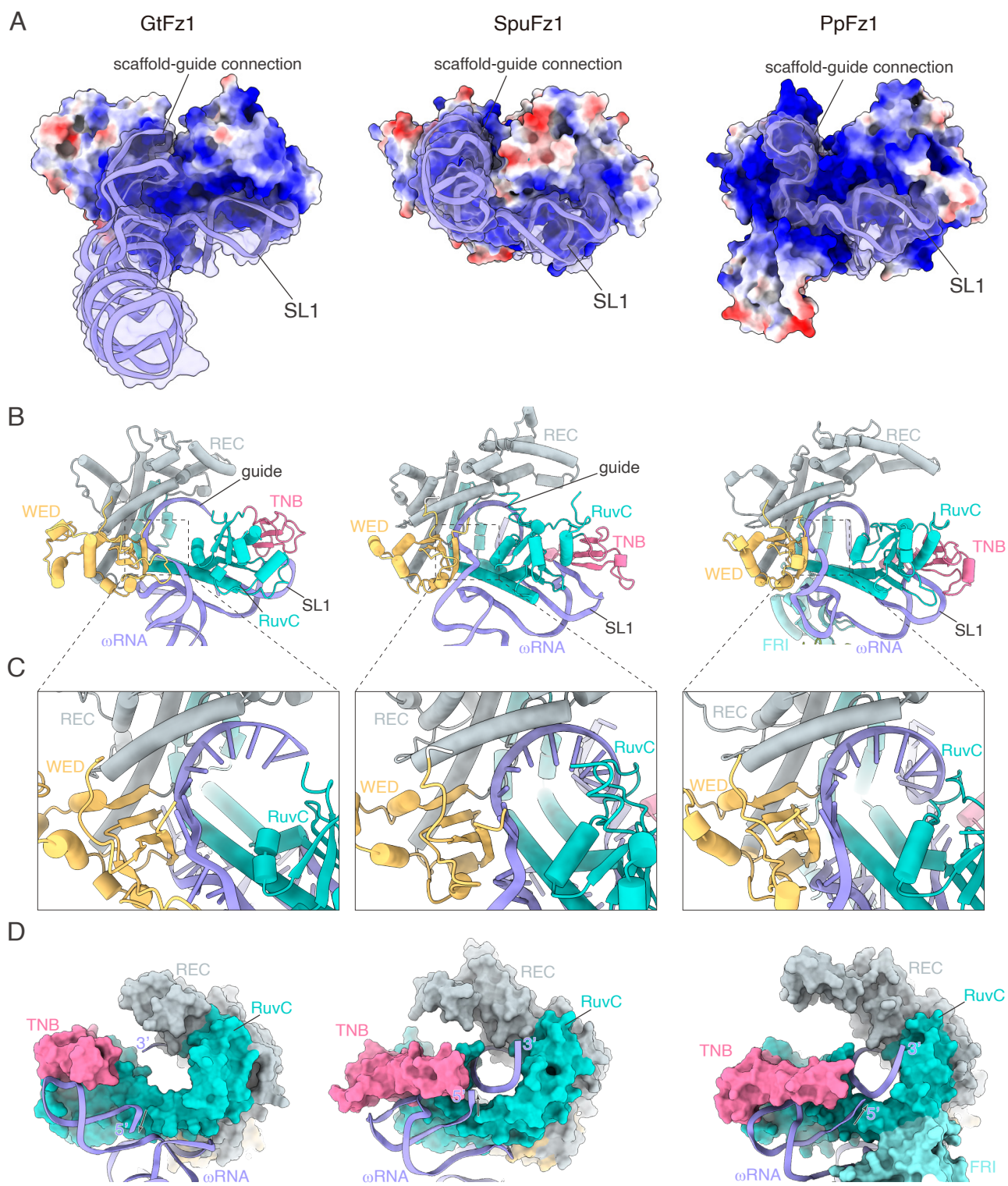

**Figure S3**

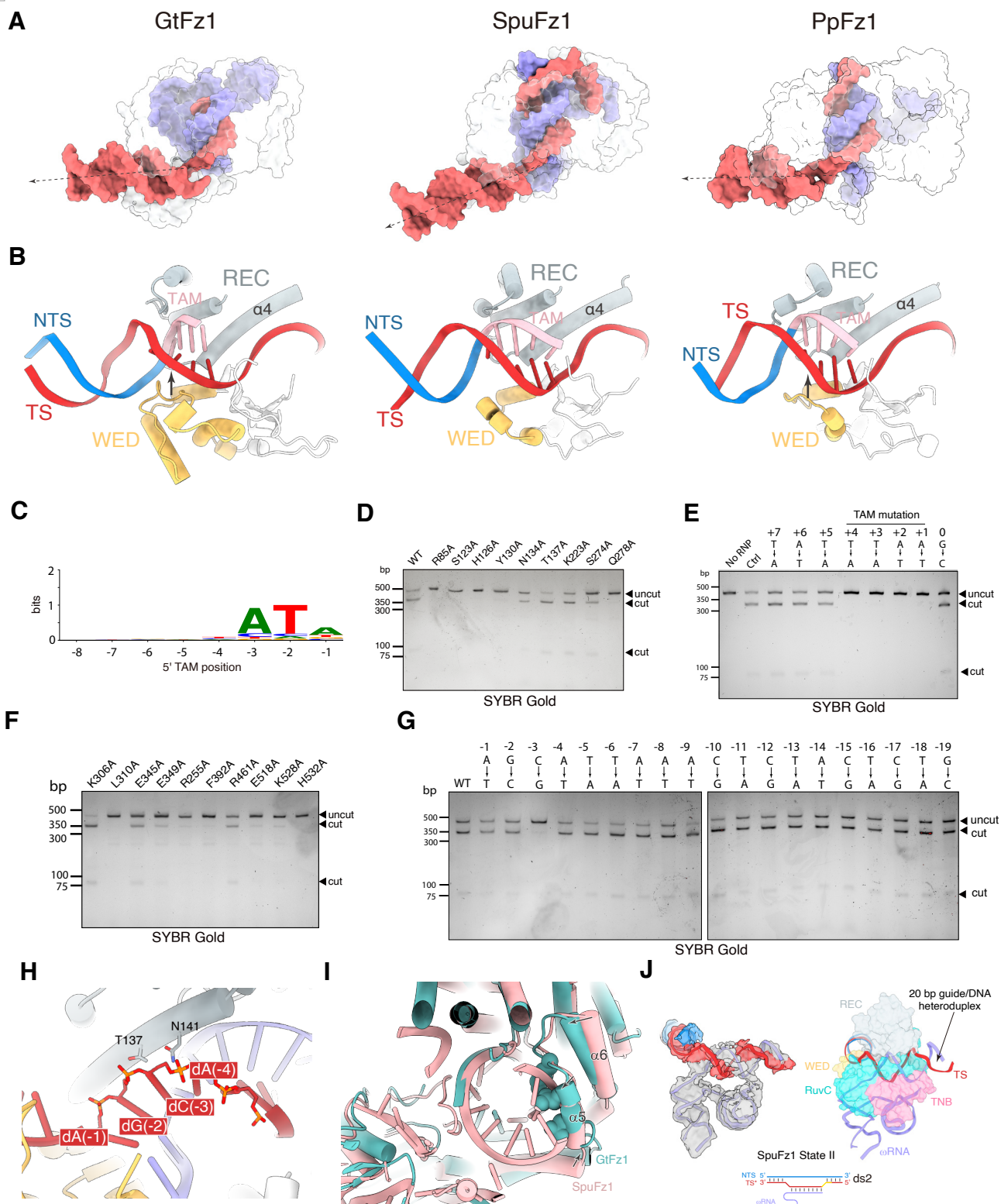

**Figure S4**

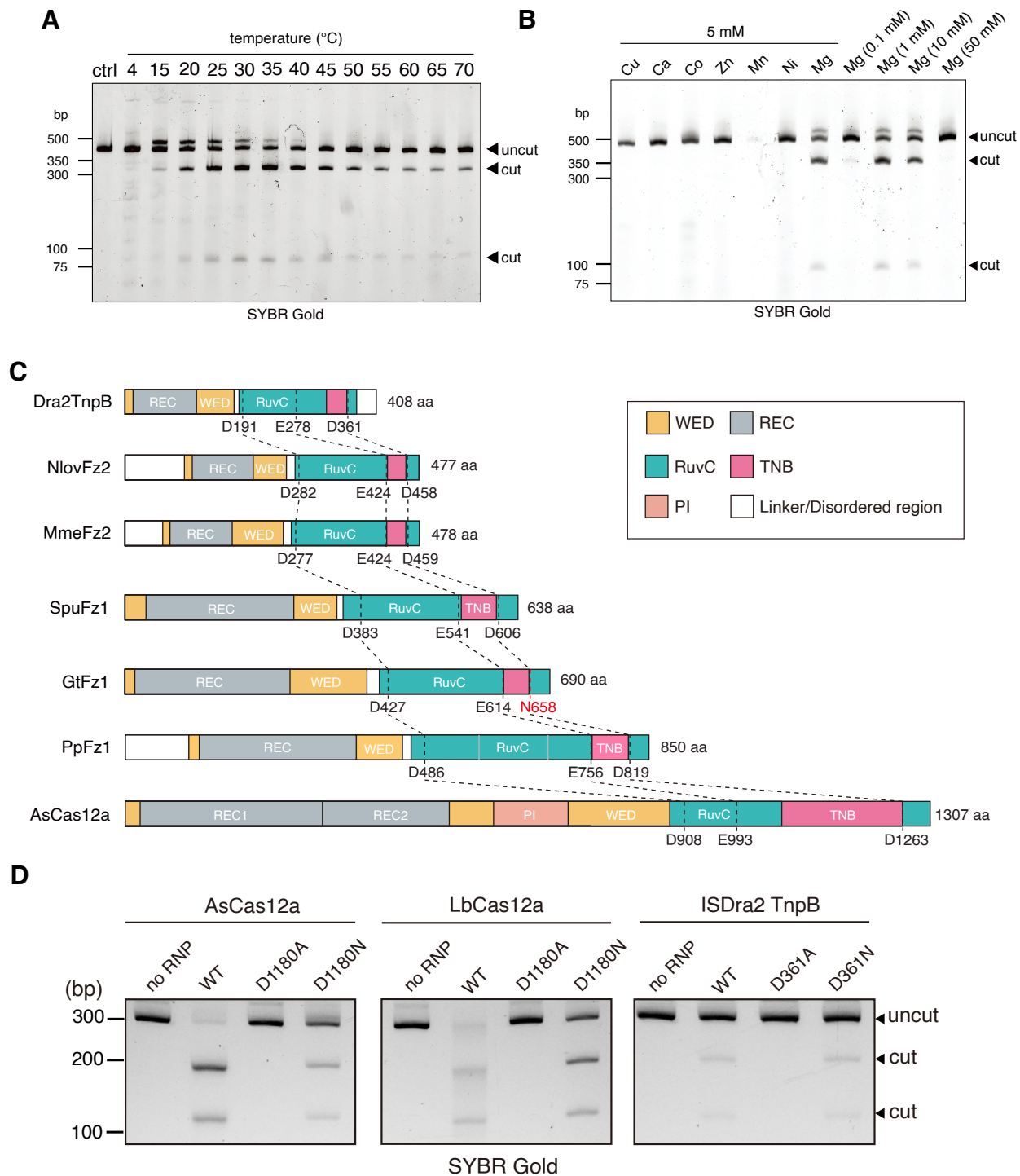

**Figure S5**

**A**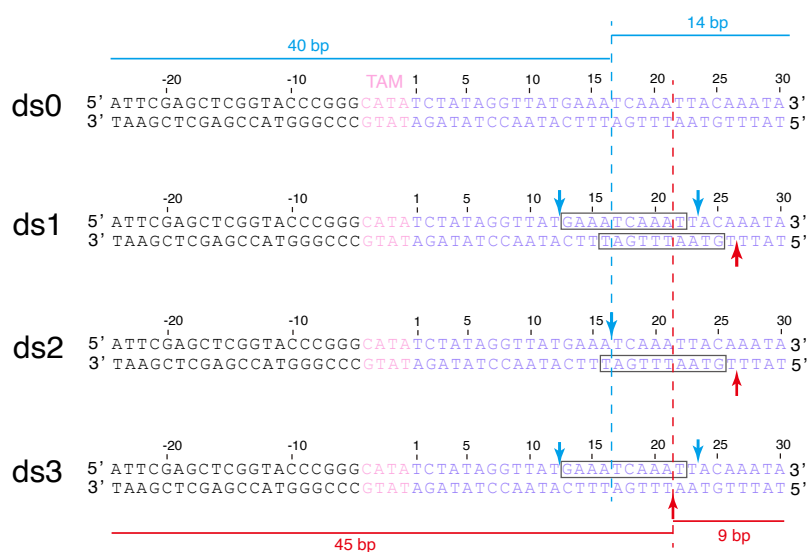**B**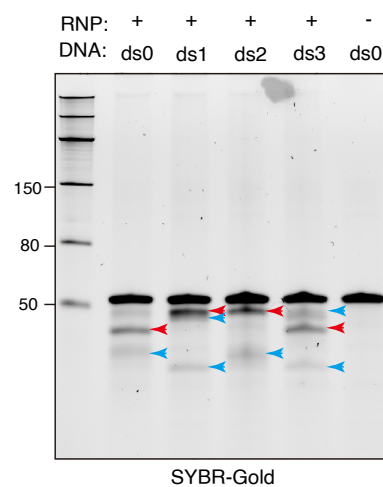**C**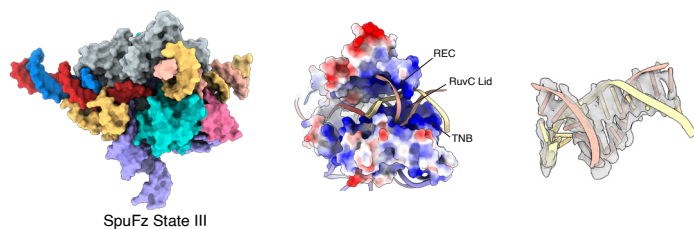**D**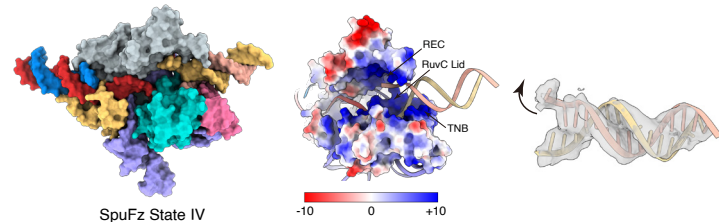**E**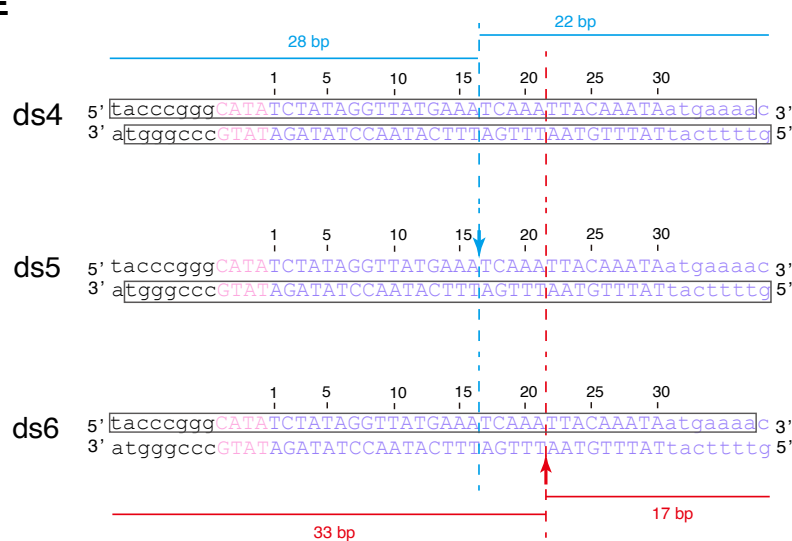**F**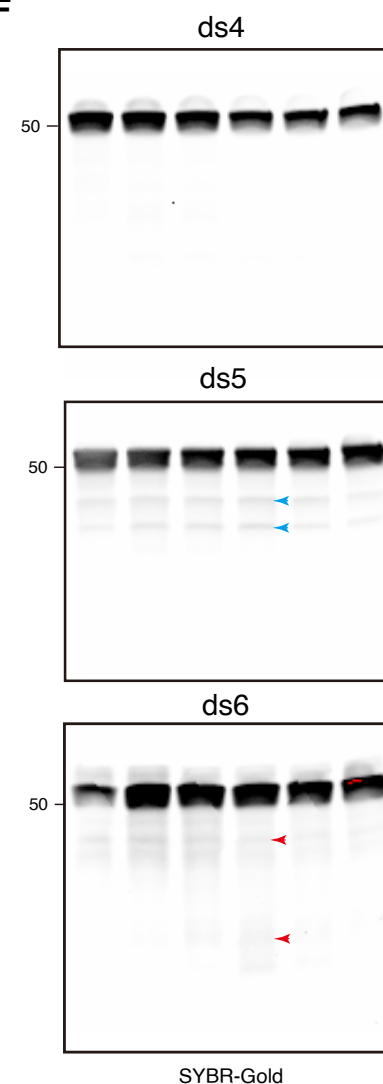**Figure S6**

**A**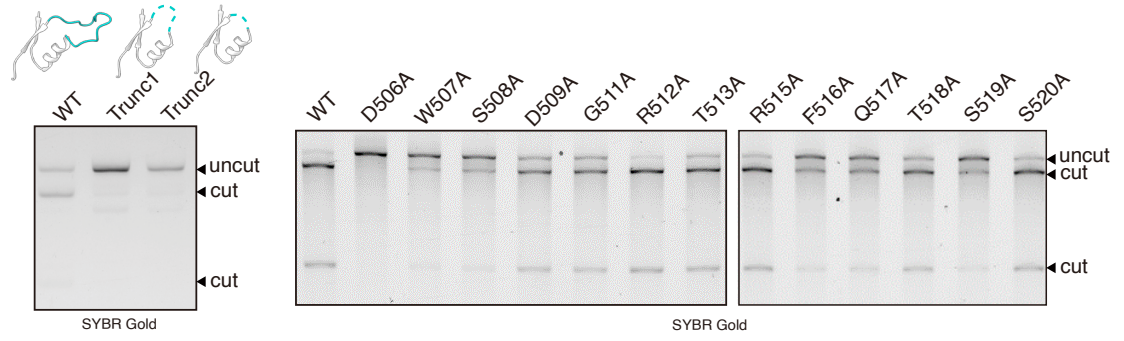**B**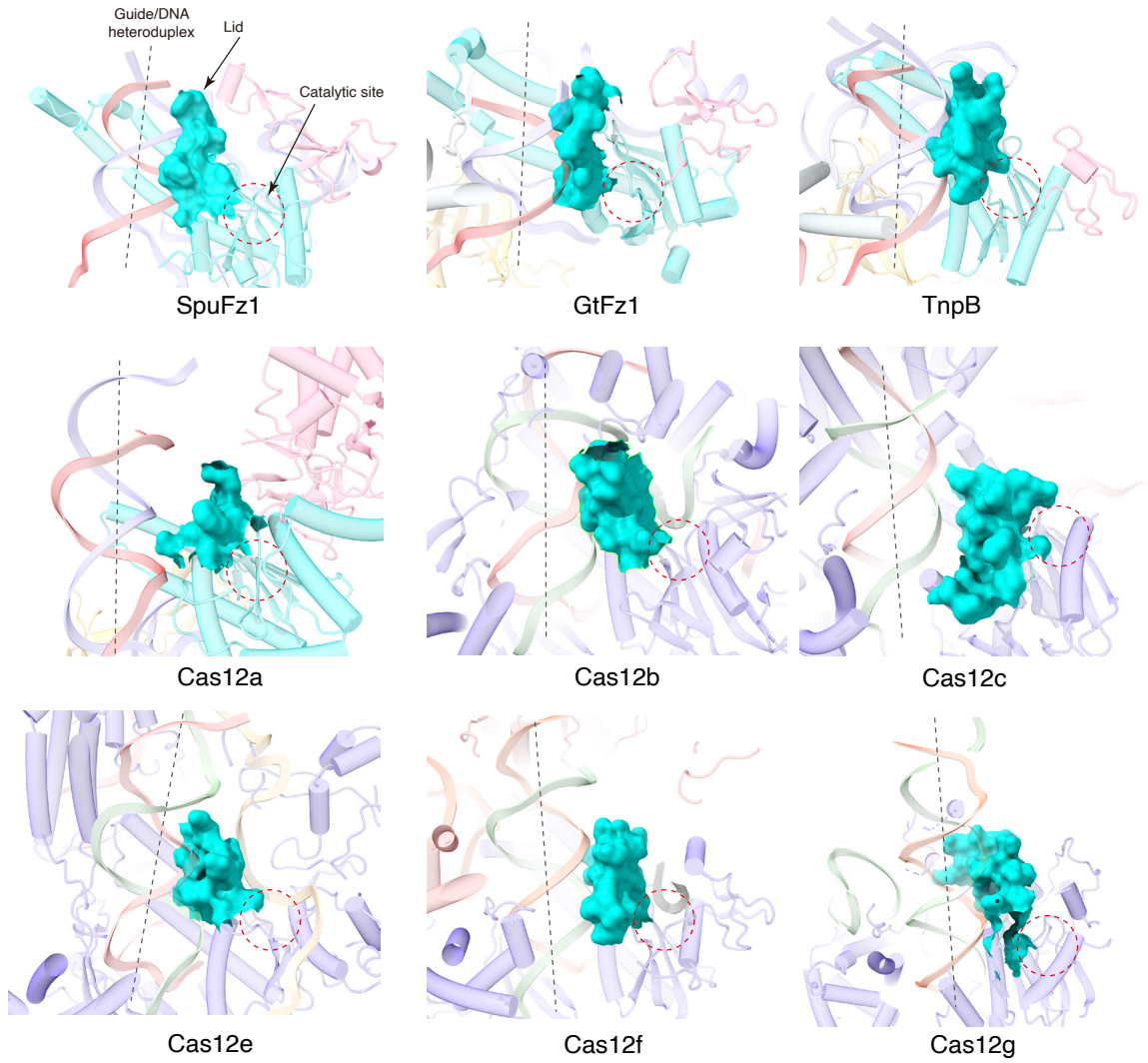**Figure S7**

**A**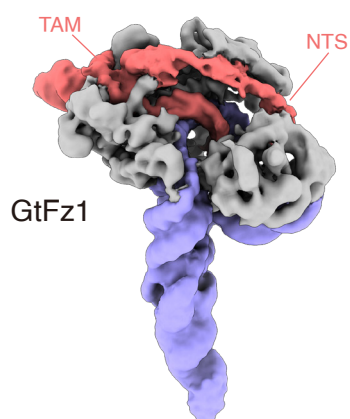**B**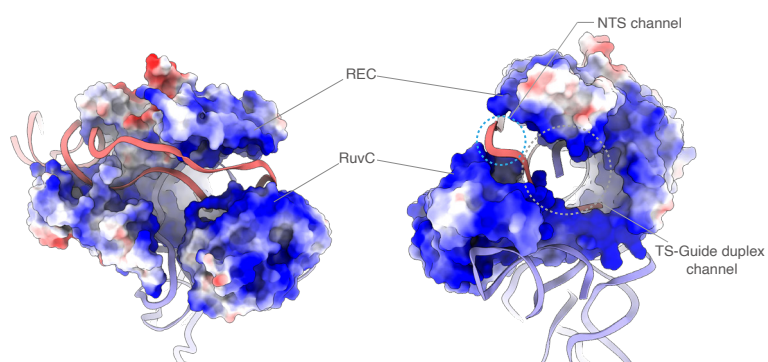**C**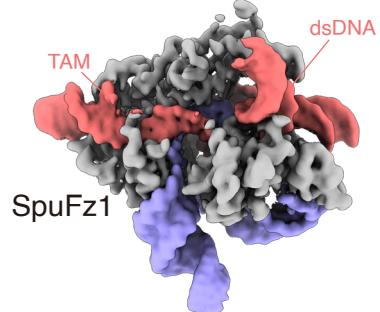**D**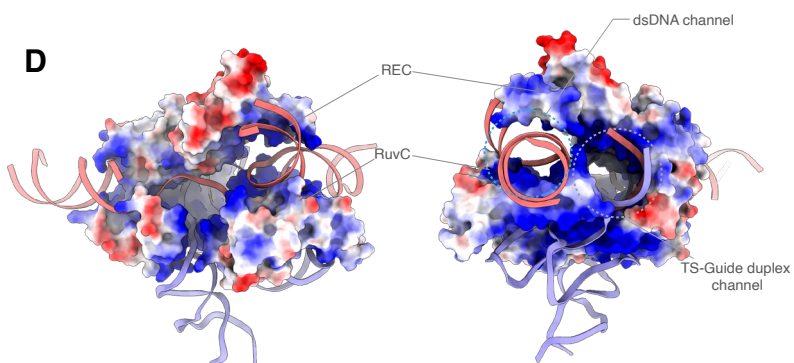**E**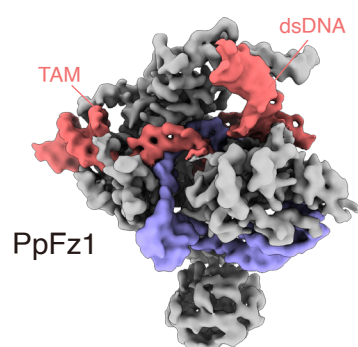**F**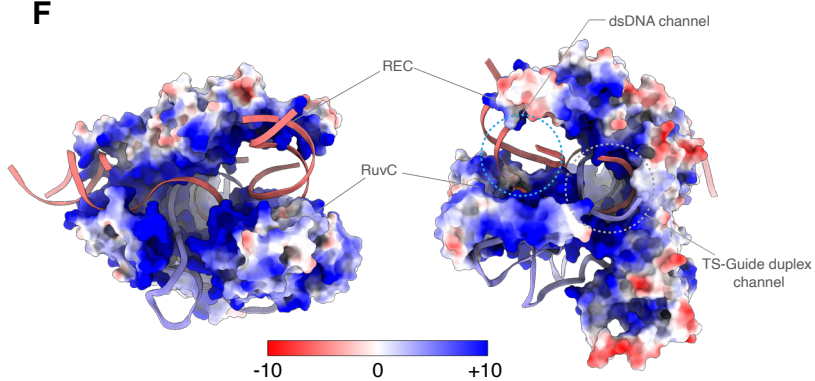**Figure S8**

Supplement: Supplemental figures — Figure S1. Cryo-EM data processing for GtFz1, SpuFz1, and PpFz1. Related to Figure 1. (A) Flow chart of cryo-EM data analysis of GtFz1 sample 1 (native guide). Two ternary complexes (State II and III) and a binary complex were obtained. (B) Flow chart of cryo-EM data analysis of GtFz1 sample 2 (PSP1 guide). The unsharpened map showing a DNA non-target strand (NTS) is stabilized by the protein. (C) Representative cryo-EM image of GtFz1 from 12,579 movies. (D) Representative and 2D averages of GtFz1 binary complex. (E) Representative and 2D averages of GtFz1 ternary complex (State III). (F) The ‘gold-standard’ FSC curves of the GtFz1 binary complex. (G) The ‘gold-standard’ FSC curves of the GtFz1 ternary complex (State III). (H) Flow chart of cryo-EM data analysis of SpuFz1. ds2: DNA target strand (TS) is partially modified and NTS is not modified. ds3: DNA TS is not modified and NTS is partially modified. ds5: DNA TS is fully modified and NTS is not modified. ds6: DNA TS is not modified and NTS is fully modified. The sequences of DNA substrate is shown in Figure S6. (I) Representative cryo-EM image of SpuFz1-ds2 from 5,628 movies. (J) Representative and 2D averages of SpuFz1. (K) Flow chart of cryo-EM data analysis of PpFz1. (L) Representative cryo-EM image of PpFz1 from 7,678 movies. (M) Representative and 2D averages of PpFz1. Figure S2. The structure of PpFz1 in complex with yeast cyclophilin. Related to Figure 1. (A) Flow chart to identify the unknown density in the PpFz1 structure. (B) Model of PpFz1 in complex with ScCyp1. (C) Interface between PpFz and ScCyp1. (D) Interaction between PpFz and ScCyp1. (E) Pull-down experiment for FRI domain with 11 cyclophilin homologs. (F) Sequence alignment of the 11 cyclophilins, including four homologs from Saccharomyces cerevisiae (ScCyp1, ScCyp2, ScCyp3, and ScCyp5), four homologs from Parasitella parasitica (PpCyp1, PpCyp2, PpCyp3, and PpCyp4), and three homologs from humans (hCypA, hCypF, and hCypH) Figure S3. Structura [file NIHMS2021671-supplement-Supplemental_figures.pdf]
