# Supplementary material for: Structural Insights into the Diversity and DNA Cleavage Mechanism of Fanzor
Source: Cell. Author manuscript; Available in PMC 2024 Sep 25. (PMC11423790; doi:10.1016/j.cell.2024.07.050)
Supplement: Table S1 — Cryo-EM data collection, refinement validation statistics. Related to Figure 1. [file NIHMS2021671-supplement-Table_S1.pdf]

Table S1. Cryo-EM data collection, refinement and validation statistics. Related to Figure 1

	**GtFz1-State I** (EMDB-45516) (PDB 9CER)	**GtFz1-State II** (EMDB-45517) (PDB 9CES)	**GtFz1-State III** (EMDB-45518) (PDB 9CET)	**SpuFz1-State I** (EMDB-45519) (PDB 9CEU)	**SpuFz1-State II** (EMDB-45520) (PDB 9CEV)
**Data collection and processing**					
Magnification	130,000	130,000	130,000	105,000	105,000
Voltage (kV)	300	300	300	300	300
Electron exposure (e–/Å^2)	59.28	59.28	59.28	48.01	48.54
Defocus range (μm)	-0.8 to -2.2	-0.8 to -2.2	-0.8 to -2.2	-0.5 to -2.6	-0.5 to -2.6
Pixel size (Å)	0.633	0.633	0.825	0.825	0.825
Symmetry imposed	C1	C1	C1	C1	C1
Initial particle images (no.)	2,014,153	2,014,153	1,455,905	7,851,258	7,851,258
Final particle images (no.)	10,507	468,829	328,609	47,576	31,906
Map resolution (Å)	4.7	3.28	3.00	3.29	3.26
FSC threshold	0.143	0.143	0.143	0.143	0.143
Map resolution range (Å)	2.5-5.0	2.5-5.0	2.5-5.0	2.5-5.0	2.5-5.0
**Refinement**					
Initial model used	AlphaFold	AlphaFold	AlphaFold	8GKH	8GKH
Model resolution (Å)					
Map sharpening method	DeepEMhancer	DeepEMhancer	DeepEMhancer	DeepEMhancer	DeepEMhancer
Model composition					
Non-hydrogen atoms	8,041	8,838	9,108	7,295	8,407
Protein residues	612	612	612	607	600
Nucleotide residues	194	181	142	108	166
Ligands	0	0	0	1	3
B factors (Å^2)					
Protein	97.62	86.64	97.62	175.96	124.35
Nucleotide	79.49	13.34	79.49	256.56	355.84
Ligand	NA	NA	NA	155.34	64.11
R.m.s. deviations					
Bond lengths (Å)	0.013	0.013	0.013	0.003	0.002
Bond angles (°)	1.990	2.090	2.081	0.765	0.584
Validation					
MolProbity score	1.06	1.08	0.94	1.11	1.29
Clashscore	1.03	1.01	0.64	3.21	5.34
Poor rotamers (%)	0.00	0.91	0.00	0.18	0.00
Ramachandran plot					
Favored (%)	97.99	95.86	96.52	98.01	99.16
Allowed (%)	2.01	4.14	3.48	1.99	0.84
Disallowed (%)	0.00	0.00	0.00	0.00	0.00

Table S1. Cryo-EM data collection, refinement and validation statistics. Related to Figure 1 (Continued)

	**SpuFz1-State III** (EMDB-45521) (PDB 9CEW)	**SpuFz1-State IV** (EMDB-45522) (PDB 9CEX)	**SpuFz1-State V** (EMDB-45523) (PDB 9CEY)	**SpuFz1-State VI** (EMDB-45524) (PDB 9CEZ)
**Data collection and processing**				
Magnification	105,000	105,000	105,000	105,000
Voltage (kV)	300	300	300	300
Electron exposure (e–/Å^2)	48.01	48.71	49.40	48.32
Defocus range (μm)	-0.5 to -2.6	-0.5 to -2.6	-0.5 to -2.6	-0.5 to -2.6
Pixel size (Å)	0.825	0.825	0.825	0.825
Symmetry imposed	C1	C1	C1	C1
Initial particle images (no.)	7,851,258	1,243,815	1,240,493	851,391
Final particle images (no.)	193,966	34,628	345,331	227,222
Map resolution (Å)	2.88	3.27	3.22	3.41
FSC threshold	0.143	0.143	0.143	0.143
Map resolution range (Å)	2.5-5.0	2.5-5.0	2.5-5.0	2.5-5.0
**Refinement**				
Initial model used	8GKH	8GKH	8GKH	8GKH
Model resolution (Å)				
Map sharpening method	b-factor -60	b-factor	DeepEMhancer	DeepEMhancer
Model composition				
Non-hydrogen atoms	7,695	7,694	7,573	8,103
Protein residues	607	600	600	600
Nucleotide residues	129	131	125	151
Ligands	3	3	1	3
B factors (Å^2)				
Protein	106.43	98.49	106.43	98.49
Nucleotide	208.49	79.49	208.49	79.49
Ligand	59.29	30.00	59.29	30.00
R.m.s. deviations				
Bond lengths (Å)	0.010	0.002	0.002	0.003
Bond angles (°)	1.699	0.629	0.482	0.632
Validation				
MolProbity score	1.07	1.38	1.33	1.40
Clashscore	2.82	6.91	6.54	7.22
Poor rotamers (%)	0.00	0.00	0.00	0.18
Ramachandran plot				
Favored (%)	98.67	98.99	98.66	98.83
Allowed (%)	1.33	1.01	1.34	1.17
Disallowed (%)	0.00	0.00	0.00	0.00

Table S1. Cryo-EM data collection, refinement and validation statistics. Related to Figure 1 (Continued)

	**PpFz1-State I** (EMDB-45525) (PDB 9CF0)	**PpFz1-State II** (EMDB-45526) (PDB 9CF1)	**PpFz1-State III** (EMDB-45527) (PDB 9CF2)	**PpFz1-State IV** (EMDB-45528) (PDB 9CF3)
**Data collection and processing**				
Magnification	105,000	105,000	105,000	105,000
Voltage (kV)	300	300	300	300
Electron exposure (e–/Å^2)	48.47	48.47	48.47	48.47
Defocus range (μm)	-0.8 to -2.3	-0.8 to -2.3	-0.8 to -2.3	-0.8 to -2.3
Pixel size (Å)	0.825	0.825	0.825	0.825
Symmetry imposed	C1	C1	C1	C1
Initial particle images (no.)	3,535,752	3,535,752	3,535,752	3,535,752
Final particle images (no.)	15,797	14,604	18,003	16,430
Map resolution (Å)	3.47	3.52	3.15	3.20
FSC threshold	0.143	0.143	0.143	0.143
Map resolution range (Å)	2.5-5.0	2.5-5.0	2.5-5.0	2.5-5.0
**Refinement**				
Initial model used	AlphaFold Model	AlphaFold Model	AlphaFold Model	AlphaFold Model
Model resolution (Å)				
Map sharpening method	DeepEMhancer	DeepEMhancer	DeepEMhancer	DeepEMhancer
Model composition				
Non-hydrogen atoms	8,869	8,934	9,733	9,263
Protein residues	888	888	888	888
Nucleotide residues	79	82	121	98
Ligands	0	0	0	0
B factors (Å^2)				
Protein	98.91	98.91	98.91	98.91
Nucleotide	79.49	79.49	79.49	79.49
Ligand	NA	NA	NA	NA
R.m.s. deviations				
Bond lengths (Å)	0.002	0.002	0.002	0.002
Bond angles (°)	0.537	0.559	0.535	0.557
Validation				
MolProbity score	1.25	1.47	1.20	1.32
Clashscore	4.75	5.19	4.23	5.29
Poor rotamers (%)	0.00	0.00	0.00	0.00
Ramachandran plot				
Favored (%)	98.19	96.83	98.75	97.85
Allowed (%)	1.70	3.17	1.25	2.15
Disallowed (%)	0.00	0.00	0.00	0.00
