# Supplementary material for: Structural Insights into the Diversity and DNA Cleavage Mechanism of Fanzor
Source: Cell. Author manuscript; Available in PMC 2024 Sep 25. (PMC11423790; doi:10.1016/j.cell.2024.07.050)
Supplement: Supplemental materials [file NIHMS2021671-supplement-Supplemental_materials.pdf]

Methods S1. Script used for the unbiased matching of the yeast proteome to the target electron microscopy density map, related to Figure 1, Figure S2, and STAR Methods.

```
#!/bin/bash

# Load necessary modules or set the environment
export PATH=/path/to/situs/bin:$PATH

# Assuming EM density map file is fixed and stored in a variable
EM_MAP="path/to/your/em_map.mrc" # Specify your EM map file path

# Directory containing PDB files
PDB_DIR="path/to/pdb_files" # Specify your directory of PDB files

# Output directory
OUTPUT_DIR="path/to/output" # Specify your desired output directory
mkdir -p ${OUTPUT_DIR}

# Count the total number of PDB files and initialize processed file count
TotalFiles=$(ls -1 ${PDB_DIR}/*.pdb | wc -l)
FilesProcessed=0

for pdb_file in ${PDB_DIR}/*.pdb; do
   FilesProcessed=$((FilesProcessed+1))
   pdb_base=$(basename "$pdb_file" .pdb)
   mkdir -p ${OUTPUT_DIR}/${pdb_base}

   # Run colores for docking and direct output to the created directory
   colores ${EM_MAP} ${pdb_file} -res 5.0 -nprocs 10 -out ${OUTPUT_DIR}/${pdb_base}/

   # Update the status for each processed file
   echo "Files processed: $FilesProcessed / $TotalFiles. Output at
${OUTPUT_DIR}/${pdb_base}/"
done

echo "Script completed. Total files processed: $TotalFiles"
```
